# *APOE ε4* associates with increased risk of severe COVID-19, cerebral microhaemorrhages and post-COVID mental fatigue: a Finnish biobank, autopsy and clinical study

**DOI:** 10.1186/s40478-021-01302-7

**Published:** 2021-12-23

**Authors:** Samu N. Kurki, Jonas Kantonen, Karri Kaivola, Laura Hokkanen, Mikko I. Mäyränpää, Henri Puttonen, FinnGen, Juha Martola, Minna Pöyhönen, Mia Kero, Jarno Tuimala, Olli Carpén, Anu Kantele, Olli Vapalahti, Marjaana Tiainen, Pentti J. Tienari, Kai Kaila, Johanna Hästbacka, Liisa Myllykangas

**Affiliations:** 1 Molecular and Integrative Biosciences and Neuroscience Center (HiLIFE), University of Helsinki, Helsinki, Finland; 2 Department of Pathology, University of Helsinki, Helsinki, Finland; 3 Department of Pathology, HUS Diagnostic Center, Helsinki University Hospital, POB 21, 00014 Helsinki, Finland; 4 Translational Immunology, Research Programs Unit, University of Helsinki, Helsinki, Finland; 5 Department of Neurology, Helsinki University Hospital, Helsinki, Finland; 6 Department of Psychology and Logopedics, Faculty of Medicine, University of Helsinki, Helsinki, Finland; 7 Department of Radiology, Medical Imaging Center, University of Helsinki and Helsinki University Hospital, Helsinki, Finland; 8 Department of Medical and Clinical Genetics, University of Helsinki, Helsinki, Finland; 9 Department of Clinical Genetics, HUS Diagnostic Center, Helsinki University Hospital, Helsinki, Finland; 10 Department of Infectious Diseases, Meilahti Infectious Diseases and Vaccine Research Center MeVac, University of Helsinki and Helsinki University Hospital, Helsinki, Finland; 11 Human Microbiome Research Program, Faculty of Medicine, University of Helsinki, Helsinki, Finland; 12 Department of Virology, University of Helsinki, and HUS Diagnostic Center, Helsinki University Hospital, Helsinki, Finland; 13 Department of Veterinary Biosciences, University of Helsinki, Helsinki, Finland; 14 Department of Neurology, University of Helsinki and Helsinki University Hospital, Helsinki, Finland; 15 Division of Intensive Care, Department of Anaesthesiology, Intensive Care and Pain Medicine, Intensive Care Unit, University of Helsinki and Helsinki University Hospital, Haartmaninkatu 4, P.O. Box 340, 00029 Helsinki, Finland

**Keywords:** *APOE4*, COVID-19 sequelae, Brain microhaemorrhage, Post-viral fatigue, Neuropathology, SARS-CoV-2, Biobank

## Abstract

*Apolipoprotein E ε4* allele (*APOE4*) has been shown to associate with increased susceptibility to SARS-CoV-2 infection and COVID-19 mortality in some previous genetic studies, but information on the role of *APOE4* on the underlying pathology and parallel clinical manifestations is scarce. Here we studied the genetic association between *APOE* and COVID-19 in Finnish biobank, autopsy and prospective clinical cohort datasets. In line with previous work, our data on 2611 cases showed that *APOE4* carriership associates with severe COVID-19 in intensive care patients compared with non-infected population controls after matching for age, sex and cardiovascular disease status. Histopathological examination of brain autopsy material of 21 COVID-19 cases provided evidence that perivascular microhaemorrhages are more prevalent in *APOE4* carriers. Finally, our analysis of post-COVID fatigue in a prospective clinical cohort of 156 subjects revealed that *APOE4* carriership independently associates with higher mental fatigue compared to non-carriers at six months after initial illness. In conclusion, the present data on Finns suggests that *APOE4* is a risk factor for severe COVID-19 and post-COVID mental fatigue and provides the first indication that some of this effect could be mediated via increased cerebrovascular damage. Further studies in larger cohorts and animal models are warranted.

**Supplementary Information:**

The online version contains supplementary material available at 10.1186/s40478-021-01302-7.

## Introduction

*Apolipoprotein E ε4* allele (*APOE4*) is the strongest genetic risk factor for sporadic Alzheimer’s disease (AD), and it has also been linked to increased risk of other neurodegenerative conditions, such as dementia with Lewy bodies and Parkinson disease dementia [1, 2]. In addition, *APOE4* has been shown to associate with increased susceptibility to SARS-CoV-2 infection and COVID-19 mortality in the UK Biobank Cohort [3, 4], and associations between *APOE4* and COVID-19 have also been reported in some other candidate gene studies [5–8]. Interestingly, recent studies have revealed that similar genetic pathways are involved in AD and severe COVID-19 [9], also in direct linkage to *APOE4 * [10]. Furthermore, these diseases share many co-morbidities and risk factors, such as age, gender, hypertension, diabetes and obesity [11].

Damage to the cerebral microvasculature has been suggested to be a central mediator of the neurological complications often seen in hospitalised COVID-19 patients [11]. Similarly, blood–brain barrier (BBB) dysfunction and cerebrovascular pathology have been shown to be involved in *APOE4*-associated neurodegenerative conditions [12]. However, information on the role of *APOE4* in COVID-19-associated pathology and parallel clinical manifestations remains scarce.

Here we studied the association between *APOE* and COVID-19 in FinnGen (https://www.finngen.fi/en) utilising the COVID-19 susceptibility GWAS release, and extended the work to include autopsy brain tissues and a prospective clinical cohort of Finnish COVID-19 patients. The Finnish population is a genetically advantageous target to further assess initial genetic discoveries because the frequency of *APOE4* is high, and its associations with related morbidity, such as AD and vascular pathology are exceptionally strong in Finland [13, 14].

## Materials and methods

### Ethics statement

Patients and controls in FinnGen provided informed consent for biobank research, based on the Finnish Biobank Act (https://www.finngen.fi/fi). All DNA samples and data were pseudonymised. Participants of the RECOVID-study [protocol approved by Ethical board of Helsinki University Hospital (HUS/1949/2020)] provided written informed consent. Clinical autopsies were performed at the Department of Pathology, HUS Diagnostic Center, Helsinki University Hospital according to Finnish legislation, with consent granted by the next of kin. The protocol covering COVID-19 autopsies was approved by the Ethics Committee of Helsinki University Hospital (HUS/1238/ 2020).

### FinnGen biobank resource

We extracted COVID-19 cases and controls from FinnGen (Release (R) 7, cutoff date March 27th 2021), which comprises prospective epidemiological cohorts (initiated in 1992), disease-based cohorts, and hospital biobank samples. All cases were extracted based on the same criteria: cases were FinnGen samples who according to the National Infectious Disease Register had had COVID-19 and from whom information on disease severity (as per level of care: home, hospital ward or intensive care unit) was available. The genotype data is integrated via a unique national personal identification number with national registries such as the hospital discharge registry, national death registry and medication reimbursement registry. Information on COVID-19 is piped into FinnGen via the national register of infectious diseases. FinnGen R7 included 3469 individuals who had a diagnosis of COVID-19. We excluded 858 cases lacking information on possible hospitalisation. We divided the remaining 2,611 cases into three groups based on the level of care as a surrogate for the severity of disease: home-isolation, hospitalisation but no intensive care, and intensive care. We selected controls from the large nationwide population-based cohorts (FINRISK1992-2012 [15], FINHEALTH2017 [16], HEALTH2000/2012 [17]) included in FinnGen. These cohorts included 43,884 individuals from whom we excluded five individuals without phenotype data and 340 individuals who had had COVID-19, leaving 43,539 individuals as controls. Then we used the MatchIt v.4.1.0 package [18] as implemented in R [19] to match all three case groups (at-home-treatment, hospitalisation, intensive care) separately to controls. Matching was performed based on age (age at death/loss-of-follow up or current age) and sex using default parameters, except that five controls were matched to every case. In a complementary analysis, we added a composite phenotype variable of any cardiovascular disease (FinnGen R7 phenotype code FG_CVD that includes hypertensive diseases, ischaemic and other heart diseases, pulmonary embolism, cerebrovascular diseases, diseases of arteries/arterioles/capillaries, diseases of veins/lymphatic vessels and lymph nodes, and other cardiovascular diseases) as an additional matching term.

*APOE* genotype was derived from the single-nucleotide polymorphisms rs429358 and rs7412 that were genotyped in FinnGen using a microarray or imputed using a Finnish reference panel. The *APOE ε1/ε3* genotype is indistinguishable from the *ε2/ε4* genotype but since the *ε1* is exceedingly rare, the *ε2/ε4* genotype was used for all individuals with the above allele combination.

### Recovery after critical COVID-19 infection (RECOVID) study design and subjects

Participants of the RECOVID-study were asked for separate written informed consent for *APOE* analysis. All participants were adult (≥ 18 years of age) native Finnish-speakers. Prior major neurological disease (e.g., traumatic brain injury, dementia, stroke or Parkinson´s disease) or developmental disability were exclusion criteria. All hospitalised COVID-19 patients with a diagnosis between March 1 and December 31, 2020 were recruited within three months from hospital discharge by mailed invitation or personal contact during a follow-up visit. Home-isolated patients with confirmed SARS-CoV-2 infection (RT-PCR or antibody test) from the same time period were recruited within three months from diagnosis by press and online announcements. Non-infected controls were recruited by online announcements in early 2021. Demographic data and blood samples were collected from all participants, and clinical data of the inpatient period was recorded from hospital-treated cohorts. One-hundred and fifty-six study participants (of whom 108 were COVID-19 patients and 48 controls) completed written questionnaires including the multi-dimensional fatigue scale (MFI-20) at six months post-discharge from hospital or recovery from acute COVID-19 (Additional file 1: Supplementary Fig. 1).

### Autopsies

Clinical autopsies were performed at the Department of Pathology, HUS Diagnostic Center according to Finnish legislation, with consent granted by the next of kin. The modified full autopsies were carried out in an autopsy room appropriate for handling infectious decedents, with appropriate personal protective equipment. All autopsies included the complete exploration of the visceral cavity and craniotomy. Modifications included extended neuropathological sampling (samples from 3–12 brain areas) in those 10 cases where a full neuropathological examination was not performed.

### Neuropathological examination

Full neuropathological examination of the brain was performed on 11 cases. Each brain was dissected after at least 10 days of formaldehyde-fixation and at least 15 samples from different brain areas were collected. H&E stainings were performed using standardised protocols at the Department of Pathology, Helsinki University Hospital.

### Immunohistochemistry (IHC)

Beta-amyloid and CD68 immunostainings were performed at the Department of Pathology, Helsinki University Hospital using standard protocols (details in Additional file 1: Supplementary Table 1).

### Histopathological analysis of perivascular haemorrhage (“Bleed Grade” and “Bleed Score”)

#### Bleed Grade

The extent of perivascular haemorrhage was graded in brain samples from midbrain, pons, medulla, cerebellum, frontal cortex and basal ganglia by two pathologists (JK, HP), reaching consensus using a consultation microscope (Nikon Eclipse i80; eye-pieces CFI 10x/22; objectives: Nikon Plan Fluor 20x/0.50, Nikon Plan Fluor 10x/0.30, Nikon Plan Fluor 4x/0.13, Nikon Plan UW 2x/0.06). Grades were defined as follows: 3 = unequivocal perivascular haemorrhage in two or more foci as seen in one field-of-view (FOV) using a 2 × objective, 2 = unequivocal perivascular haemorrhage as seen in one FOV using a 2 × objective, 1 = unequivocal perivascular haemorrhage as seen in one FOV using a 4 × objective, 0 = no unequivocal perivascular haemorrhage in any FOV as seen using a 4 × objective. The pathologists graded the samples blinded to the information on genotype.

#### Bleed Score

The number of haemorrhagic foci were determined in brain samples from midbrain, pons, medulla, cerebellum, frontal cortex and basal ganglia by one pathologist (JK) using the same microscope as in the Bleed Grade. Scoring was performed as follows: four parenchymal hot spots for haemorrhage (total FOV 95 mm^2^) were scored using the 4 × objective and counting the number of haemorrhagic foci. Perivascular haemorrhage was defined as an enlarged perivascular space with red blood cells or enlarged perivascular space with debris and pigmented macrophages. The border of the perivascular space also had to show evidence of neuropil degeneration or vacuolation. Meningeal haemorrhage was not counted. The pathologist scored the samples blinded to information on genotype.

### Analysis of cerebral amyloid angiopathy (CAA)

CAA was determined by a neuropathologist (LM) based on beta-amyloid-immunopositivity in at least one vessel on sections from frontal cortex and cerebellum.

### Histopathological analysis of microglial reactivity

CD68-stained sections from midbrain, pons and medulla were assessed by two pathologists (JK, HP) using a modified version of the method described by Poloni and colleagues [20]. Briefly, the CD68-stained slide was assessed for representative areas using a 4 × objective, and five FOVs were scored according to the 4-grade scheme using a 10 × objective (FOV 3.8 mm^2^), with confirmatory use of higher magnification where needed. The 4-point scale (0–3) used was identical to the work by Poloni: 0 = absence of both perivascular infiltrate and microglial nodules and < 20 amoeboid cells/reactive microglial cells; 1 = presence of at least one perivascular infiltrate or 1 micronodule or > 20 amoeboid cells/reactive microglial cells; 2 = presence of 2–4 microglial nodules; and 3 = presence of > 4 microglial nodules. The pathologists graded the samples blinded to information on genotype, and grade was agreed upon by consensus at the consulting microscope described above.

In addition, a count of stained foci was determined by one pathologist (JK) in 3 hot spot areas using a 20 × objective (FOV 0.95 mm^2^). Where a single cell could be identified, the stained cells were counted. The pathologist scored the samples blinded to information on genotype.

### Sanger sequencing

We genotyped *APOE* using Sanger sequencing. All consented participants of the RECOVID study gave venous blood samples from which we extracted DNA using standard methods. In the AUTOPSY cohort, DNA was isolated from frozen tissues collected at autopsy using standard protocols. We amplified DNA fragments containing rs429358 and rs7412 using previously published primers [21] that amplify rs429358 and rs7412 on the same DNA fragment. The amplification reaction contained 1X DreamTaq Buffer, 0.35 mM of dNTP mix, 0.6 μM of forward and reverse primers, 1 M of Betaine, 1.25U of DreamTaq DNA Polymerase (Applied Biosystems, ThermoFisher Scientific, Waltham, MA, USA), approximately 50 ng of template DNA and nuclease-free water to attain a total volume of 20 μl. The cycling conditions have been previously published [21]. We then purified the amplicons using Exonuclease I—Shrimp Alkaline Phosphatase Clean Up (Applied Biosystems) according to the manufacturer’s protocol. We then sequenced the purified amplicons using BigDye Terminator v3.1 Cycle sequencing kit (Applied Biosystems) according to the manufacturer’s protocol with the forward primer. Sequences were analysed on a ABI3730XL DNA Analyzer (Applied Biosystems) at the Finnish Institute of Molecular Medicine (FIMM). We determined the genotypes of rs429358 and rs7412 using Sequencher (Sequencher® version 5.1 DNA sequence analysis software, Gene Codes Corporation, Ann Arbor, MI USA).

### Fatigue testing (Multidimensional Fatigue Inventory MFI-20)

We evaluated chronic fatigue at a 6-month follow-up using the Multidimensional Fatigue Inventory (MFI-20) score [22]. The questionnaire was translated into Finnish and was a part of a larger set of questions concerning neuropsychological recovery and mental health. MFI-20 measures five subscales of fatigue (General Fatigue, Physical Fatigue, Mental Fatigue, Reduced Motivation, Reduced Activity), each of which is probed with four questions. The subject specifies the extent to which each statement relates to her/him on a 5-point Likert scale, ranging from “Yes, that is true” to “No, that is not true”. The range of possible scores is 4–20 in each fatigue subscale, and 20–100 in total fatigue, with a higher score signifying a higher level of fatigue.

### Statistical analysis of the AUTOPSY and RECOVID cohorts

All statistical analyses were performed using GraphPad Prism (version 8.4.2 for Windows, GraphPad Software, San Diego, California USA, www.graphpad.com), SPSS (IBM Corp. Released 2020. IBM SPSS Statistics for Windows, Version 27.0. Armonk, NY: IBM Corp), and the statistical software R [19].

We used Fisher’s exact test as implemented in R’s stats package to test if carrying at least one *APOE ε4* allele associated with each COVID-19 case group. We used linear mixed-effect model as implemented in R’s nlme (Pinheiro J, Bates D, DebRoy S, Sarkar D, R Core Team (2021). nlme: Linear and Nonlinear Mixed Effects Models. R package version 3.1–153, https://CRAN.R-project.org/package=nlme) and cumulative link mixed model as implemented in R’s ordinal (Christensen, R. H. B. (2019). ordinal—Regression Models for Ordinal Data. R package version 2019.12–10, https://CRAN.R-project.org/package=ordinal) package to determine the statistical significances of perivascular bleed score and bleed grade depending on *APOE4* carriership, respectively. Additionally, we used exact Pearson’s chi-square test and Mann–Whitney U test as implemented in SPSS to determine the statistical significance of perivascular bleed grade and bleed score depending on *APOE4* carriership specifically in different brain areas, respectively. All patients who filled in the MFI-20 questionnaire were included in the analysis. We used a negative binomial regression model as implemented in the MASS [23] package in R to analyse factors associated with the MFI-20 fatigue score in both univariate and multivariate analyses (Tables 2 and 3). Negative binomial regression was chosen over Poisson regression to compensate for the overdispersion in data. COVID-19 severity was classified into three categories, as done in a previously published analysis of fatigue associated with long-COVID [24]. The categories were: 0 – noninfected controls, 1 – home-isolated COVID-19, 2 – hospitalised COVID-19. As COVID-19 severity was the only non-binary categorical variable in multivariate binomial regression model and reached statistical significance in the model in one but not all factor levels, its independence was further analysed by comparison (ANOVA) with the same model with severity excluded.

## Results

**Fig. 1:**
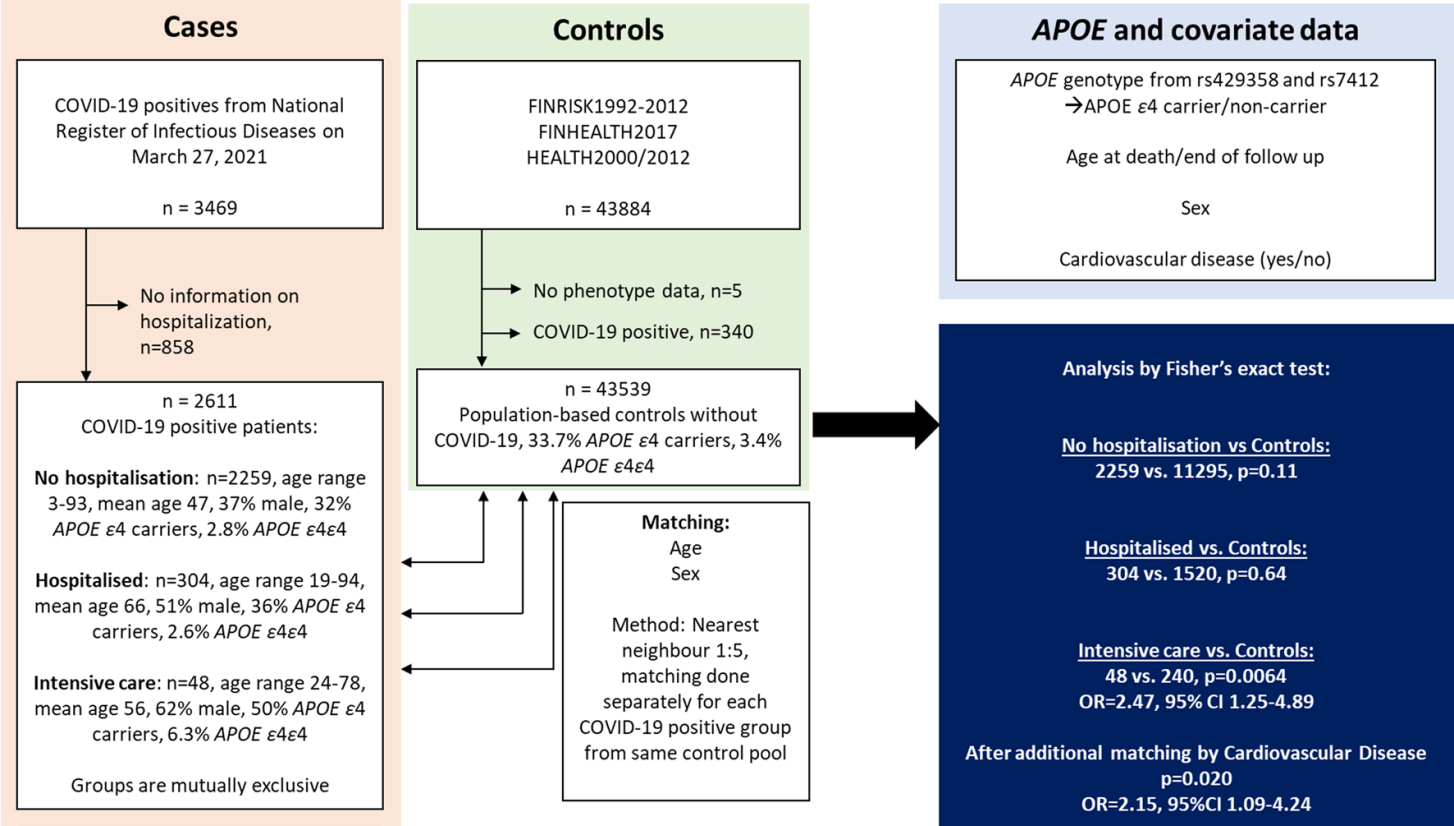
Flow chart and results of FinnGen Release 7 data analysis

To assess the association between *APOE* and COVID-19, we used data from FinnGen release 7, which integrates genome-wide genotype data of over 300,000 subjects with phenotype data derived from national registries. First, we identified 2611 individuals with a COVID-19 diagnosis in the National Registry of Infectious diseases by March 27th 2021 and with known disease-severity: home-isolated (n = 2259, 31.8% *APOE4* carriers); hospitalised but no intensive care (n = 304, 36.2% *APOE4* carriers); and intensive care (n = 48, 50.0% *APOE4* carriers). In a complementary analysis, we matched five controls from nationwide population-based cohorts to every case by age and sex, and also by diagnosis of cardiovascular disease (Fig. 1). Compared to the matched controls, *APOE4* carriership was not significantly more frequent in the home-isolated (*p* = 0.11, odds ratio (OR) = 0.92, 95% confidence interval (CI) 0.84–1.02, Fisher’s exact test) nor hospital treated COVID-19 patients (*p* = 0.64, OR = 1.07, 95% CI 0.82–1.39, Fisher’s exact test). However, *APOE4* carriership was more prevalent among COVID-19 patients treated in the intensive care unit (ICU) (*p* = 0.0064, OR = 2.47, 95% CI 1.25–4.89, Fisher’s exact test), and the association persisted also after matching for cardiovascular disease (*p* = 0.020, OR = 2.15, 95% CI 1.09 – 4.24, Fisher’s exact test).

Next, we looked into the prevalence of *APOE4* in two COVID-19 cohorts, which we sequenced for *APOE* genotype status, and matched the genotypes with histological results from neuropathological autopsy material, or clinical records, respectively. The cohorts were (1) a series of autopsies comprising 21 cases with COVID-19 infection confirmed by RT-PCR (group “AUTOPSY”, see Methods and Additional file 1: Supplementary Table 2), and (2) a prospective cohort study of Recovery After Critical COVID-19 Infection (RECOVID). The RECOVID cohort comprised 58 patients treated for critical COVID-19 in the intensive care unit (ICU) in Helsinki University Hospital, and 33 COVID-19 patients admitted at ward-level care (WARD). In line with our main aim to study the impact of COVID-19 severity, the ICU and WARD cohorts were compared to 37 COVID-19 patients who did not need hospitalisation during recovery (HOME), and 53 non-infected individuals (NOCOV; see Additional file 1: Supplementary Fig. 1 and Methods for more details on the study design). The demographic and *APOE4* data of both cohorts are shown in Table 1.

**Table 1:** Characteristics of the study cohorts used in *APOE* genotyping

	RECOVID					AUTOPSY
	Group NOCOV	Group ICU	Group WARD	Group HOME	All COVID-19 + (ICU & WARD & HOME)	Group AUTOPSY
	% (n)	% (n)	% (n)	% (n)	% (n)	% (n)
	N = 53	N = 58	N = 33	N = 37	N = 128	N = 21
Female Sex	49% (26)	47% (27)	67% (22)	73% (27)	59% (76)	33% (7)
Age, median (IQR)	56 (50–64)	63.5 (55.25–69.75)	59 (53–65)	46 (37–55)	58 (47–66)	71 (59–71)
Age categories						
21–40 years	9% (5)	3% (2)	6% (2)	35% (13)	13% (17)	10% (2)
41–60 years	58% (31)	31% (18)	52% (17)	49% (18)	41% (53)	19% (4)
> 60 years	32% (17)	66% (38)	42% (14)	16% (6)	45% (58)	71% (15)
BMI, median (IQR)	26.9 (23.3–28.9)^a^	29.8 (27.3–32.1)	29.0 (24.8–33.1)^b^	26.1 (23.4–28.5)^c^	28.6 (25.0–31.6)	
Any comorbidity	32% (17)	81% (47)	79% (26)	49% (18)	71% (91)	
Asthma	0% (0)	16% (9)	30% (10)	5% (2)	16% (21)	
Hypertension	13% (7)	50% (29)	33% (11)	14% (5)	35% (45)	81% (17)
Diabetes	2% (1)	28% (16)	6% (2)	3% (1)	15% (19)	19% (4)
Chronic Heart Disease	2% (1)	19% (11)	3% (1)	11% (4)	13% (16)	
Hypercholesterolemia	11% (6)	33% (19)	12% (4)	3% (1)	19% (24)	
Current or prior smoker^d^		48% (28)	30% (10)			
*APOE ε*4 carrier	39.6% (21)	34.5% (20)	42.4% (14)	37.8% (14)	37.5% (48)	29% (6)
*APOE ε*4*ε*4 genotype	5.7% (3)	3.4% (2)	9.1% (3)	8.1% (3)	6.3% (8)	4.8% (1)

^a^Missing data for six patients;

^b^Missing data for one patient;

^c^Missing data for 14 patients;

^d^Data only available from hospitalised patients

*APOE4* carriership was not associated with severity of COVID-19 in the small AUTOPSY or RECOVID cohorts (Table 1). For the AUTOPSY group, we used the FinnGen population as controls (p = 0.49, Fisher’s exact test). In the RECOVID cohort, we compared the various severity grades of the disease to the non-infected group (ICU vs NOCOV, *p* = 0.69; WARD vs NOCOV *p* = 0.82, HOME vs NOCOV *p* = 1, Fisher’s exact test in all comparisons).

**Fig. 2:**
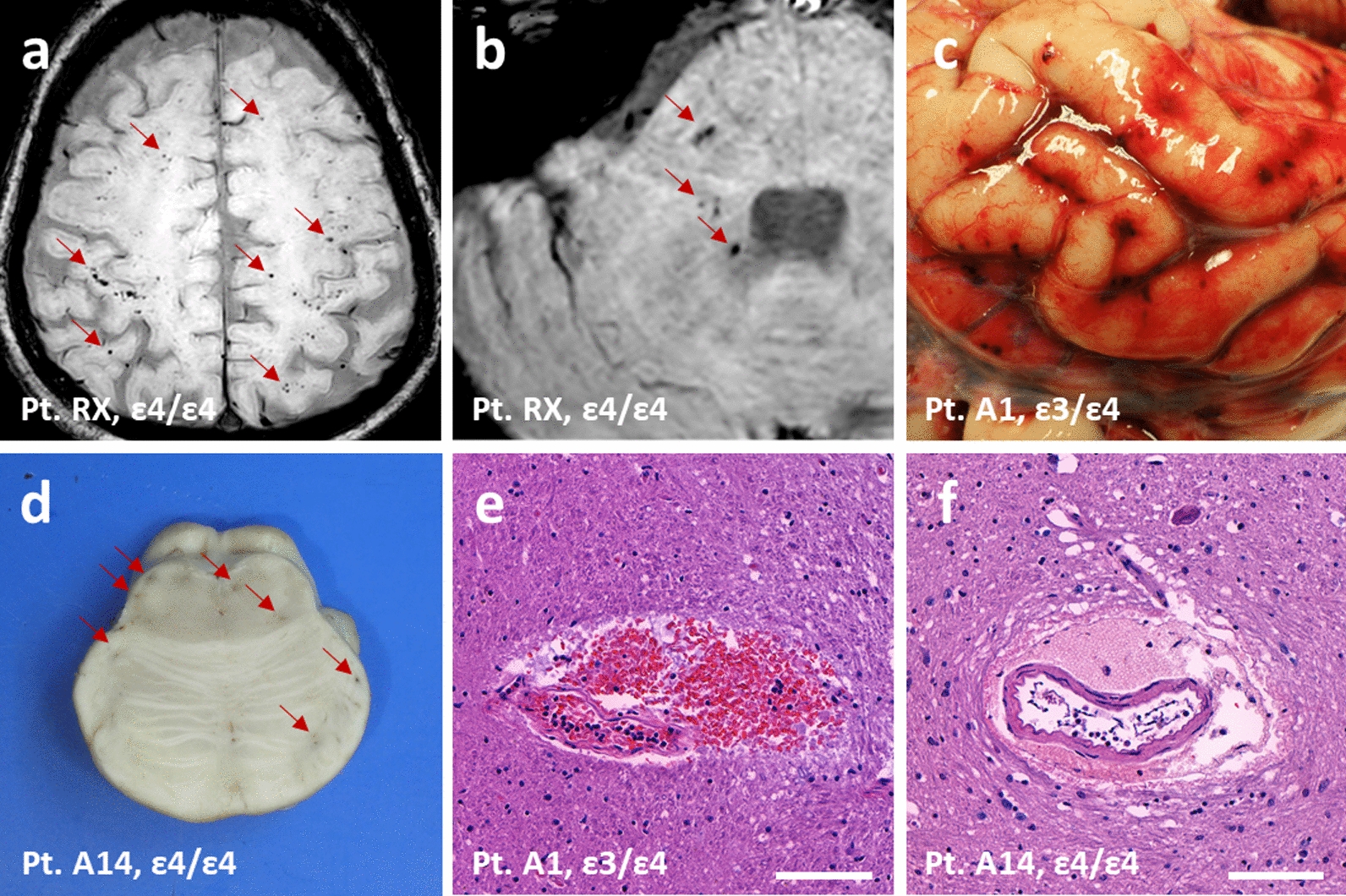
Microvascular brain haemorrhages in RECOVID and AUTOPSY cohorts. **a** MRI showing cortical microhaemorrhages in a case with *APOE ε4/ε4*. **b** MRI showing cerebellopontine microhaemorrhages in the same case as (**a**). **c** Brain subarachnoidal microhaemorrhages at autopsy in a case with *APOE ε3/ε4*.** d** Brain pontine microhaemorrhages at neuropathological examination in a case with *APOE ε4/ε4*. **e** Histological H&E section showing pontine microhaemorrhages in the same case as (**c**). **f** Histological H&E section showing pontine microhaemorrhages in the same case as (**d**). Red arrows indicate microhaemorrhages. Scale bars represent 100 μm in (**e**) and (**f**)

Interestingly, we found that AUTOPSY and RECOVID cohorts included *APOE4* homozygotes (cases RX and A14; Fig. 2) sharing a striking pathological phenotype: the presence of abundant petechial microvascular haemorrhages in multiple brain areas confirmed by either brain MRI or neuropathological autopsy. The clinical features of RX are described in detail in the Supplementary Information. The neuropathological and clinical features of A14 have been described in detail previously [25]. Here, we also performed whole-genome sequencing of this patient (findings of the main COVID-19-associated variants are shown in Additional file 1: Supplementary Table 3), in addition to the *APOE* analysis. Microvascular brain injury has been reported to associate with COVID-19 [26], and autopsied COVID-19 patients have been reported to exhibit micro- and perivascular haemorrhages [27, 28] (but see also [29]). However, we are not aware of any reports associating it with the *APOE* genotype status. Therefore, we set out to look closer at brain microvascular damage in the AUTOPSY cohort.

**Fig. 3:**
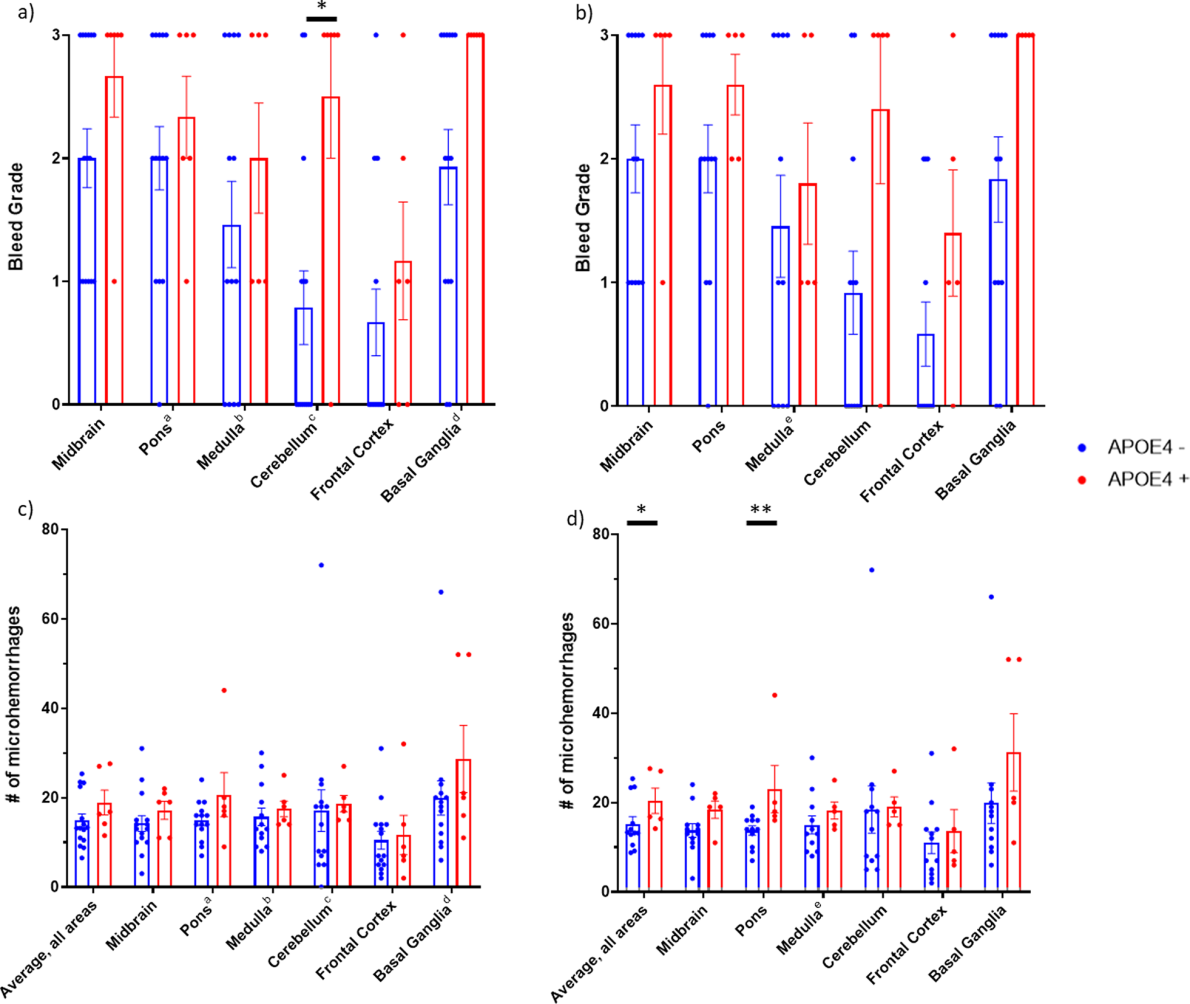
Presence of microvascular haemorrhages across brain areas. **a** Bleed Grades of all autopsied cases (n = 21). **b** Bleed Grades with *APOE2* carriers excluded (n = 17). **c** Bleed Scores of all autopsied cases.** d** Bleed Scores with* APOE2* carriers excluded. ^a,c,d,e^Missing sample from 1 patient; ^b^Missing samples from 2 patients. * stands for nominal* p* < 0.05 (non-significant after Bonferroni’s correction for multiple testing), ** stands for nominal* p *< 0.01 (significant after Bonferroni’s correction for multiple testing)

We examined histopathological sections from different brain areas of all 21 subjects of the AUTOPSY cohort, using haematoxylin–eosin (H&E) staining and IHC stainings. The severity of perivascular haemorrhage was assessed with a 4-grade scoring system (see Bleed Grade in Methods) and a continuous bleed score for the frequency of perforating microvascular damage (see Bleed Score in Methods) in six brain areas (frontal cortex, mesencephalon, pons, basal ganglia, cerebellum and medulla). *APOE4* carriers had a significantly higher bleed grade than non-carriers overall (*p* = 0.0024, cumulative link mixed model), with the most pronounced effect in cerebellum (nominal *p* = 0.024, Pearson’s chi-square test). Basal ganglia showed a similar trend (nominal *p* = 0.062) with all of the *APOE4* carriers having the most severe grade. With respect to the bleed score, there was no statistically significant effect of *APOE4* carriership (*p* = 0.201, linear mixed-effect model) over the whole dataset. In both analyses, and in each brain area, the mean value for bleed grade/score was higher in *APOE4* carriers (Fig. 3 and Additional file 1: Supplementary Table 4).

A recent mechanistic study on *APOE4*-related cerebrovascular disorders [30] did not include *APOE ε2* (*APOE2*) -containing genotypes, and the role of *APOE2* as a protective versus predisposing factor is under debate and seems to vary according to condition [31]. Therefore, we further analysed the microhaemorrhage data excluding *APOE2* carriers. In this analysis, the effect of *APOE4* to the bleed grade persisted (*p* = 0.0053). In bleed scores, there was still no statistically significant difference when assessing the data as a whole (*p* = 0.114), but in brain area -specific comparisons microhaemorrhages in pons were significantly more prevalent in *APOE4* carriers (*p* = 0.007, significant after correction with Bonferroni’s method for multiple testing) (Fig. 3).

**Fig. 4:**
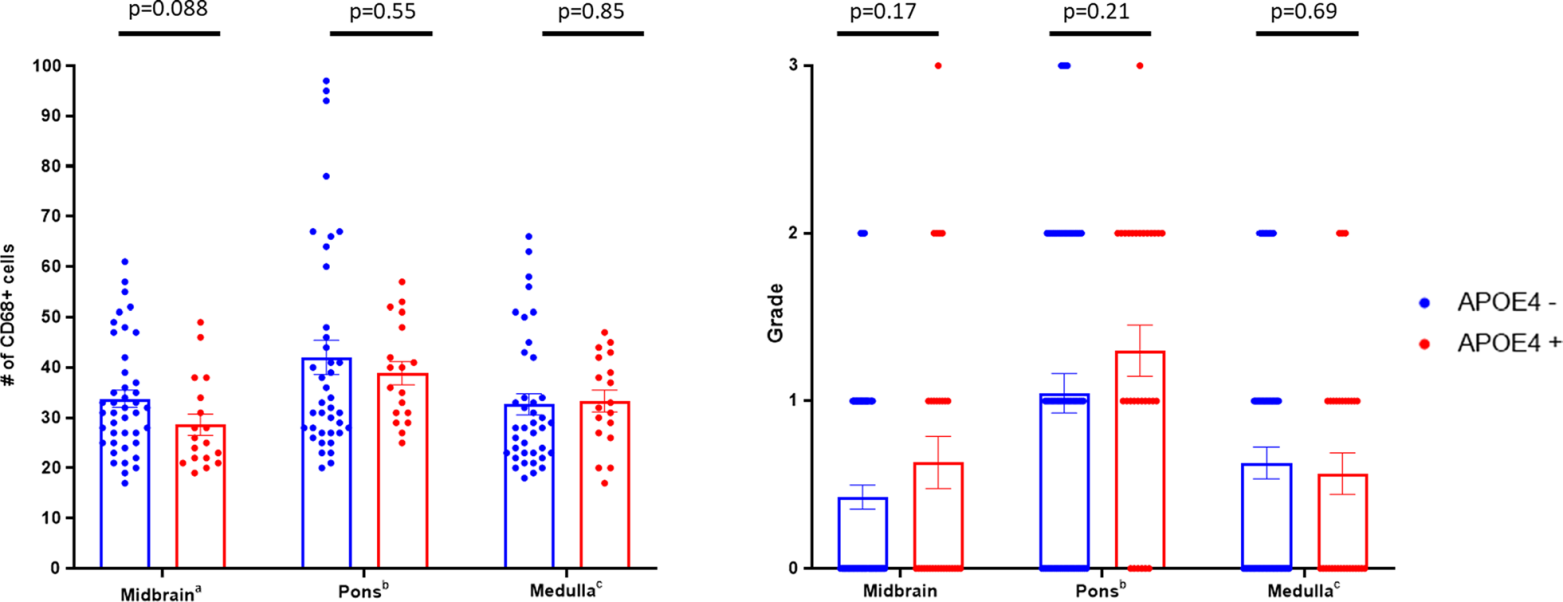
Abundance and pro-inflammatory reactivity of CD68 + microglial cells across brain areas. **a** The number of CD68-positive cells in evaluated brain areas; three FOVs evaluated per area from each autopsied case (n = 21). **b** Grade of microglial activation in evaluated brain areas, as described in [20]; five FOVs scored per area from each autopsied case (n = 21). *P *values calculated with unpaired t-test. ^a^Missing sample from 1 patient; ^b,c^Missing sample from 2 patients

Perivascular activated (CD68-positive) microglia in the brainstem have previously been reported as a common finding in COVID-19 patients [32]. As *APOE4*-associated neuroinflammatory effects are known to be partially mediated by microglia [33], we also tested whether microglial activation was associated with *APOE4* carriership in COVID-19; however, there was no such association (Fig. 4, *p* > 0.05 in all studied brain areas).

Chronic fatigue has been recently reported [24, 34, 35] as a prevalent symptom in post-acute COVID-19 syndrome (PACS, or “long COVID”). Similar observations were also published after the severe acute respiratory syndrome (SARS) epidemic in 2003 [36]. As *APOE4* strongly associates with various kinds of cognitive dysfunctions, we also analysed its potential role on the long-term neuropsychological recovery after COVID-19, using MFI-20 fatigue scores (validated for post-viral syndrome in adults [37]) six months post-discharge. In a previous report, which showed increased post-COVID fatigue after severe initial illness at a 6-month follow-up [24], the analysis was stratified to hospitalised and home-isolated patients. To enable a direct comparison with the above study, we combined the ICU and WARD groups into one cohort of hospitalised patients. However, we found that *APOE4* carriership was not associated with total MFI-20 scores, nor with the subcategories of general fatigue, physical fatigue, reduced activity or reduced motivation (Table 2).

**Table 2:** Fatigue scores at 6-month follow-up by *APOE4* carrier status – negative binomial regression analysis

	NOCOV (n = 48)			HOME (n = 33)			Hospitalised (ICU&WARD) (n = 75)		
	*APOE ε4* –	*APOE ε4* +	OR (95% CI) *P* value	*APOE ε4* –	*APOE ε4* +	OR (95% CI) *P* value	*APOE ε4* –	*APOE ε4* +	OR (95% CI) *P* value
	n = 30	n = 18		n = 20	n = 13		n = 48	n = 27	
	Mean	Mean		Mean	Mean		Mean	Mean	
Total Fatigue^a^	37.8	41.7	1.10 (0.93–1.31) 0.258	53.4	53.9	1.01 (0.78–1.31) 0.941	50.2	53.5	1.07 (0.91–1.25) 0.434
General Fatigue^b^	8.0	9.3	1.15 (0.93–1.43) 0.191	12.6	11.8	0.93 (0.73–1.20) 0.593	12.3	12.7	1.04 (0.87–1.24) 0.696
Physical Fatigue^b^	7.3	8.1	1.11 (0.90–1.37) 0.336	11.1	12.0	1.08 (0.81–1.42) 0.604	11.8	11.9	1.00 (0.83–1.20) 0.987
Mental Fatigue^b^	7.4	9.8	1.32 (1.08–1.63) **0.0062**	11.2	10.8	0.96 (0.68–1.35) 0.821	8.0	9.9	1.23 (1.02–1.49) **0.029**
Reduced Motivation^b^	6.8	6.1	0.90 (0.71–1.14) 0.389	8.2	8.1	0.99 (0.70–1.41) 0.96	8.5	8.3	0.98 (0.80–1.19) 0.845
Reduced Activity^b^	8.4	8.4	1.01 (0.79–1.28) 0.94	10.3	11.3	1.10 (0.81–1.50) 0.556	9.6	10.7	1.12 (0.93–1.34) 0.245

Analysis of the effect of *APOE4* status on mental fatigue was done by negative binomial regression. OR, odds ratio. Statistical significance at the level < 0.05 is shown in bold text.

^a^Possible score from 20 (no fatigue) to 100 (highest possible fatigue);

^b^Possible score from 4 (no fatigue) to 20 (highest possible fatigue)

Notably, the subcategory of mental fatigue was associated with a higher score in hospitalised (ICU + WARD) *APOE4* carriers compared to non-carriers (9.9 vs 8.0, *p* = 0.029, negative binomial regression, Table 2), and this association remained independently significant after factoring in other plausibly contributing patient features in a multivariate model (*p* = 0.038, negative binomial regression, Table 3). *APOE4* carriership similarly elevated mental fatigue scores also in the NOCOV group (9.8 vs 7.4, *p* = 0.0062), whereas it did not have an effect in the HOME group (10.8 vs 11.2, *p* = 0.82).

**Table 3:** Factors associated with mental fatigue at 6-month follow-up—negative binomial regression analysis

Factor	Mental Fatigue^a^ in hospitalised RECOVID patients (ICU&WARD) N = 75			Mental fatigue in all RECOVID subjects N = 156		
	n (%)	Univariable models OR (CI) *P*	Multivariable model OR (CI) *P*	n (%)	Univariable models OR (CI) *P*	Multivariable model OR (CI) *P*
Female Sex	38 (51%)	1.03 (0.85–1.25) 0.74	0.96 (0.77–1.19) 0.72	87 (56%)	1.15 (1.00–1.32) 0.052	1.06 (0.92–1.24) 0.42
Older age^b^		1.00 (0.99–1.01) 0.90	1.00 (0.99–1.01) 0.94		0.992 (0.987–0.997) **0.0035**	**0.99 (0.99–1.00) 0.034**
COVID-19 severity^c^		*Not applicable*		48 (31%) – grade 0^c^	1.33 (1.10–1.60) – grade 1	1.21 (0.99–1.47) – grade 1
				33 (21%) – grade 1^c^	1.04 (0.89–1.22) – grade 2	1.02 (0.86–1.21) – grade 2
				75 (48%) – grade 2^c^	**0.0053**	0.11
COVID-19 positivity		*Not applicable*		108 (69%)	1.13 (0.97–1.31) 0.12	*By definition included into COVID-19 Severity*
APOE4 carriership	27 (36%)	1.23 (1.02–1.49) **0.029**	**1.25 (1.01–1.55) 0.038**	58 (37%)	1.19 (1.03–1.37) **0.015**	**1.19 (1.03–1.36) 0.015**
BMI^b^		1.00 (0.98–1.02) 0.83	1.00 (0.98–1.02) 1.00		*Not applicable (21 missing data points)*	
Asthma	14 (19%)	1.14 (0.90–1.45) 0.26	1.15 (0.89–1.48) 0.28	16 (10%)	1.03 (0.82–1.29) 0.78	1.06 (0.84–1.34) 0.62
Hypertension	32 (43%)	0.97 (0.80–1.18) 0.75	1.03 (0.81–1.30) 0.83	43 (38%)	0.93 (0.80–1.09) 0.39	1.03 (0.85–1.25) 0.74
Diabetes	16 (21%)	1.01 (0.80–1.28) 0.91	1.07 (0.81–1.42) 0.61	18 (12%)	1.01 (0.81–1.25) 0.95	1.16 (0.91–1.48) 0.24
Chronic Heart Disease	11 (15%)	1.00 (0.76–1.30) 1	0.93 (0.68–1.26) 0.62	15 (10%)	1.05 (0.83–1.33) 0.68	1.10 (0.86–1.41) 0.44

Analysis of associated factors on mental fatigue score was done by negative binomial regression. The analysis was performed separately in the cohort of hospitalized COVID-19 patients in the RECOVID study, and the cohort of all subjects genotyped in the RECOVID study. Statistical significance at the level < 0.05 is shown in bold text.

^a^Possible score from 4 (no fatigue) to 20 (highest possible fatigue score);

^b^N (%) not applicable to age and BMI which were analysed as continuous variables;

^c^Three-grade scale: 0 = Non-infected controls, 1 = Home-isolated COVID-19, 2 = Hospital-treated COVID-19

We further analysed several factors (sex, age, COVID-19 severity, *APOE4* carriership and key comorbidities) in relation to mental fatigue in all RECOVID participants (Table 3), and *APOE4* remained a significant contributor (*p* = 0.015 in multivariate model). The severity of COVID-19 was not associated with mental fatigue after controlling for other factors (*p* = 0.11 in multivariate model, Table 3).

## Discussion

Previous studies [3, 4] showed a genetic association between *APOE4* carriership and COVID-19 positivity, as well as mortality. Using Finnish biobank (FinnGen study), autopsy and clinical evidence, we confirm and extend these findings. First, we show an association between *APOE4* carriership and COVID-19 patients requiring critical care. Second, our data suggests that *APOE4* carriership increases the incidence of cerebral microhaemorrhages in COVID-19 patients, and that *APOE4*-associated COVID-19 neuropathology might be driven more by the perivascular damage than by microglial activation. Finally, our findings in the RECOVID cohort suggest that *APOE4* carriership may be a risk factor for prolonged mental fatigue after severe COVID-19.

Several candidate-gene studies (Additional file 1: Supplementary Table 5a) have found an association between *APOE4* and SARS-CoV-2 infection or the severity of COVID-19, whereas genome-wide association studies on COVID-19 (Additional file 1: Supplementary Table 5b) have failed to find evidence for an association between the *APOE* locus and COVID-19. There are several reasons for the discrepant results, such as different definitions for cases and controls, different age distributions and different ancestries. Candidate-gene studies may be more susceptible to false positive findings and publication bias. In genome-wide association analyses, reaching the genome-wide significance threshold requires a very large sample size or a very large effect size. The largest genome-wide association studies published were multinational and it is known that *APOE4* frequency varies in different populations. Moreover, genome-wide association studies test the association of individual variants. Thus, they did not directly test the association of *APOE4*, just the association of *APOE4* proxy variants, which may decrease power.

The populations with *APOE4* carrier frequency above the global average (like the Finns or in the UK) are ideal for studying the genetic association reported here, because the effect size of *APOE4* carriership for increased susceptibility to severe COVID-19 is modest, and therefore likely to be masked in more heterogeneous transethnic populations, such as in a recent global genetic mapping of COVID-19 [38].

The key limitations of this study are the low number of ICU-treated subjects in our biobank data and subjects overall in the RECOVID study, which was not adequately powered to detect an effect of *APOE4* on COVID-19 severity. Additionally, the association of *APOE4* with mental fatigue was not consistent, as the control group also showed it, while the HOME group did not. Selection bias could account for the finding in the NOCOV group, since the cohort was recruited via open invitation and, thus, individuals experiencing fatigue-like cognitive symptoms may have been more eager to participate. The HOME group on the other hand showed higher mental fatigue scores in general, which suggests that factors unrelated to COVID-19 could be masking an underlying effect of *APOE4*. Moreover, only the subcategory of mental fatigue (which comprises questions probing the subjects’ ability to direct attention and keep focused) differed significantly between genotypes, whereas the other dimensions of the MFI-20 questionnaire did not. Therefore, further studies with more complete test batteries are warranted to study the link of *APOE* genotype and cognitive symptoms. Limitations of our AUTOPSY cohort include the small number of patients with variable comorbidities, COVID-19 disease courses and mechanisms of death. When assessing haemorrhages, even small bleeds were taken into account. It is noteworthy that in Finland anticoagulation treatment was routinely used in COVID-19 patients even in the early stages of the pandemic, and the majority of the autopsied patients were treated with anticoagulants. Many of them suffered from hypoxia and had been treated in ICU for varying lengths of times, affecting the bleeding tendency in variable ways. Most importantly, *APOE4* is per se associated with early mortality and cognitive decline, and if and how this is modified by COVID-19 is a question that cannot be conclusively answered by this study alone.

*APOE4* is the strongest common genetic risk factor for late-onset AD, and it has also been shown to associate with cognitive decline independently of AD pathology [39]. These effects have been mechanistically connected to the *APOE4*-induced compromised cerebrovascular integrity including the breakdown of the BBB in both humans and animal models, mediated by inflammatory pathways in BBB-forming pericytes, as well as in neurons [30]. COVID-19 has also been shown to damage the microvasculature of the brain [26] and lead to enhanced expression of APOE at the barriers of the CNS: the choroid plexus and in astrocytes, which participate in formation of the BBB [40]. Interestingly, an in vitro study based on human-induced pluripotent stem cell (hiPSC) models has suggested that astrocytes and neurons expressing APOE4 homozygously are more vulnerable to SARS-CoV-2 infection than those with APOE3, and APOE4 astrocytes also exhibit a more severe cytopathogenic effect to SARS-CoV-2 infection, which might mediate the severity of COVID-19 [41]. However, direct viral involvement and neurotropism seem to be rare events in human subjects [26]. Furthermore, the effects of APOE in the context of COVID-19 are likely not restricted to the brain—for instance, APOE has a pro-inflammatory role also in the lungs [42], and *APOE4* is a well-known risk factor for atherosclerosis and organ damage outside the brain predisposing to life-threatening vicious cycles. Thus, we hypothesise that the severe multi-organ inflammatory and coagulopathic manifestations of COVID-19 are potentiated by the effects of APOE4, and therefore *APOE4* carriers might be prone to developing a more severe course of disease. Studies in larger cohorts and animal models are warranted to investigate this hypothesis.

## Conclusions

The present data on Finns suggests that *APOE4* is a risk factor for severe COVID-19 and post-COVID mental fatigue and provides the first indication that some of this effect could be mediated via increased cerebrovascular damage. Further studies in larger cohorts and animal models are warranted.

## Supplementary Information

**Additional file 1:** Supplementary Information.

## Data Availability

The datasets used and/or analysed during the current study are available from the corresponding authors upon reasonable request.

## Abbreviations

AD: Alzheimer’s disease
*APOE2*: Apolipoprotein E *ε2* allele
*APOE4*: Apolipoprotein E *ε4* allele
AUTOPSY: Cohort of autopsied cases
BBB: Blood–brain barrier
CAA: Cerebral amyloid angiopathy
FOV: Field-of-view
HOME: Cohort of COVID-19 patients who did not need hospitalisation during recovery
ICU: Intensive care unit; cohort of COVID-19 patients admitted to an intensive care unit
IHC: Immunohistochemistry
MFI-20: Multi-dimensional fatigue scale—20
NOCOV: Cohort of non-COVID-19-infected individuals
PACS: Post-acute COVID-19 syndrome
RECOVID: Recovery After Critical COVID-19 Infection
WARD: Cohort of COVID-19 patients admitted at ward-level care
