# Supplementary material for: APOE ε4 associates with increased risk of severe COVID-19, cerebral microhaemorrhages and post-COVID mental fatigue: a Finnish biobank, autopsy and clinical study
Source: Acta Neuropathol Commun. 2021 Dec 23;9:199. doi: 10.1186/s40478-021-01302-7 (PMC8696243; doi:10.1186/s40478-021-01302-7)
Supplement: Supplementary file 1 — Additional file 1: Supplementary Information. [file 40478_2021_1302_MOESM1_ESM.docx]

**Supplementary Information**

***APOE* *ε4* associates with increased risk of severe COVID-19, cerebral microhaemorrhages and post-COVID mental fatigue: a Finnish biobank, autopsy and clinical study**

**Samu N. Kurki*, Jonas Kantonen*, Karri Kaivola*, Laura Hokkanen, Mikko I. Mäyränpää, Henri Puttonen, FinnGen, Juha Martola, Minna Pöyhönen, Mia Kero, Jarno Tuimala, Olli Carpén, Anu Kantele, Olli Vapalahti, Marjaana Tiainen, Pentti J. Tienari, Kai Kaila, Johanna Hästbacka^^¤^, Liisa Myllykangas^^¤^**

* These authors contributed equally

^ These authors contributed equally

¤ Correspondence: Liisa Myllykangas, MD, PhD

Department of Pathology, University of Helsinki and HUS Diagnostic Center, Helsinki University Hospital

POB 21, 00014 Helsinki, Finland

Email: [liisa.myllykangas@helsinki.fi](mailto:liisa.myllykangas@helsinki.fi)

Johanna Hästbacka, MD, PhD

Division of Intensive Care, Department of Anaesthesiology, Intensive Care and Pain Medicine

University of Helsinki and Helsinki University Hospital

Intensive Care Unit, P.O. Box 340, Haartmaninkatu 4, 00029 HUS, Helsinki, Finland

Email: johanna.hastbacka@hus.fi

Patient RX: Brief Clinical Report

Patient RX, a person in their late sixties was admitted to hospital in spring 2020 because of SARS-CoV-2 infection confirmed by RT-PCR. The patient was an ex-smoker, had a history of essential hypertension, hypercholesterolaemia, sleep apnea, coronary artery disease, and obesity (BMI 29.8 kg/m^2^). After a brief treatment period at ward-level care, the patient was admitted to the intensive care unit (ICU) with invasive mechanical ventilation due to respiratory failure. The patient was delirious already at the time of admission to the ICU. ICU treatment included mechanical ventilation, prone positioning and intravenous corticosteroids. The patient was diagnosed with myocarditis in the ICU, and a transient conduction abnormality. After extubation, the patient had a persisting and severe delirium requiring prolonged ICU care and treatment continued at the university hospital level for three weeks after the respiratory failure had resolved. Soon after discharge, the patient contacted the post-COVID outpatient clinic reporting recurring loss of memory, and was referred to undergo neuropsychological evaluation. In autumn 2020, the patient suffered a short episode of intense headaches (which resolved without treatment), and complained of memory dysfunction. Remarkably, the patient spontaneously regained cognitive faculties to a pre-COVID level during spring 2021.

Notably, delirium has frequently been encountered among hospitalised COVID-19 patients [43], and a recent study found that APOE e4 genotypes were associated with an even higher risk of delirium during COVID-19 related hospitalisations [8].

**Supplementary Methods**

Whole-genome sequencing (case A14)

Case A14 was whole-genome sequenced from 300 ng of genomic DNA using Illumina PCR-free prep and the NovaSeq S4 XP (NovaSeq v1.5 chemistry) to produce 150 base pair pair-end reads to 40x coverage. Read processing, read mapping to GRCh38, variant calling and variant quality control was done with DRAGEN v. 3.8 pipeline (Illumina) at the Finnish Institute of Molecular Medicine.

**Supplementary References**

43. Kennedy M, Helfand BKI, Gou RY, Gartaganis SL, Webb M, Moccia JM, et al. Delirium in Older Patients With COVID-19 Presenting to the Emergency Department. JAMA Netw Open [Internet]. American Medical Association; 2020;3:e2029540–e2029540. Available from: https://jamanetwork.com/journals/jamanetworkopen/fullarticle/2773106

44. Genomewide Association Study of Severe Covid-19 with Respiratory Failure. N Engl J Med [Internet]. Massachusetts Medical Society; 2020;383:1522–34. Available from: https://www.nejm.org/doi/full/10.1056/nejmoa2020283

45. Wu P, Ding L, Li X, Liu S, Cheng F, He Q, et al. Trans-ethnic genome-wide association study of severe COVID-19. Commun Biol 2021 41 [Internet]. Nature Publishing Group; 2021;4:1–10. Available from: https://www.nature.com/articles/s42003-021-02549-5

46. Pairo-Castineira E, Clohisey S, Klaric L, Bretherick AD, Rawlik K, Pasko D, et al. Genetic mechanisms of critical illness in COVID-19. Nat 2020 5917848 [Internet]. Nature Publishing Group; 2020;591:92–8. Available from: https://www.nature.com/articles/s41586-020-03065-y

47. Ma Y, Huang Y, Zhao S, Yao Y, Zhang Y, Qu J, et al. Integrative genomics analysis reveals a 21q22.11 locus contributing risk to COVID-19. Hum Mol Genet [Internet]. Oxford University Press; 2021;30:1247. Available from: /pmc/articles/PMC8136003/

48. Shelton JF, Shastri AJ, Ye C, Weldon CH, Filshtein-Sonmez T, Coker D, et al. Trans-ancestry analysis reveals genetic and nongenetic associations with COVID-19 susceptibility and severity. Nat Genet 2021 536 [Internet]. Nature Publishing Group; 2021;53:801–8. Available from: https://www.nature.com/articles/s41588-021-00854-7

49. Mapping the human genetic architecture of COVID-19. Nat 2021 [Internet]. Nature Publishing Group; 2021;1–8. Available from: https://www.nature.com/articles/s41586-021-03767-x

50. Mousa M, Vurivi H, Kannout H, Uddin M, Alkaabi N, Mahboub B, et al. Genome-wide association study of hospitalized COVID-19 patients in the United Arab Emirates. EBioMedicine [Internet]. Elsevier; 2021;74:103695. Available from: /pmc/articles/PMC8587122/

51. Li Y, Ke Y, Xia X, Wang Y, Cheng F, Liu X, et al. Genome-wide association study of COVID-19 severity among the Chinese population. Cell Discov 2021 71 [Internet]. Nature Publishing Group; 2021;7:1–16. Available from: https://www.nature.com/articles/s41421-021-00318-6

**Supplementary Figures and Tables**


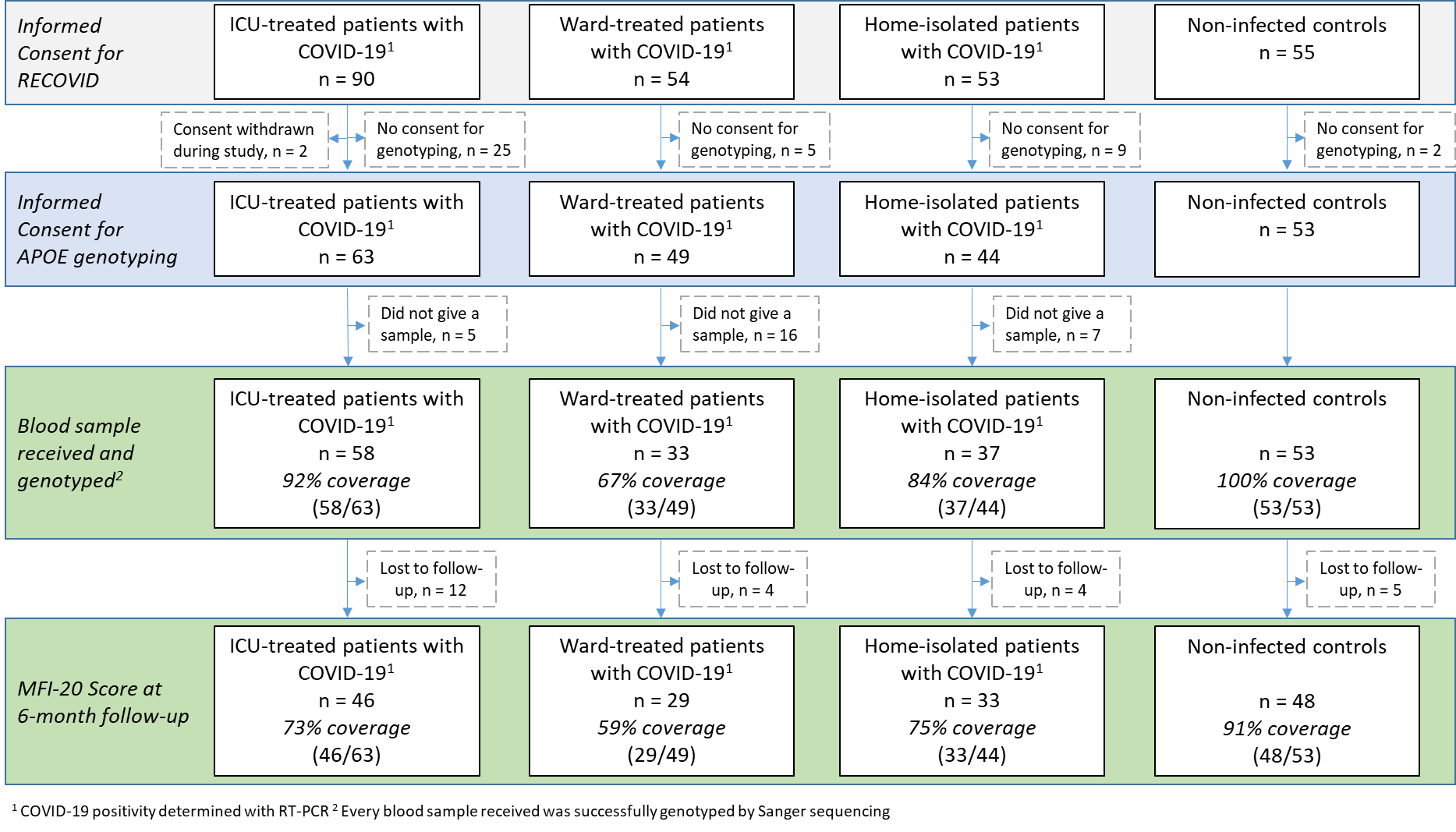
Supplementary Figure 1. RECOVID cohort *APOE*-genotyping and fatigue inventory

Supplementary Table 1. Antibody information


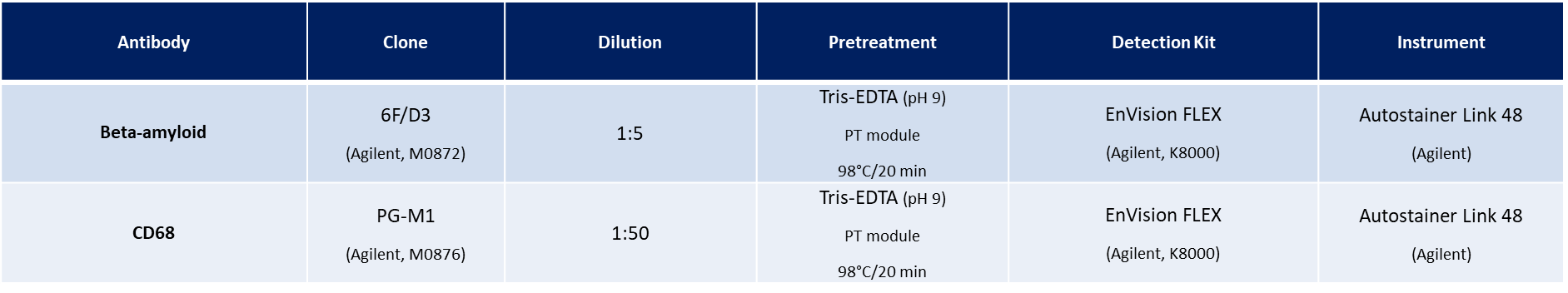


Supplementary Table 2. Characteristics of the autopsied cases


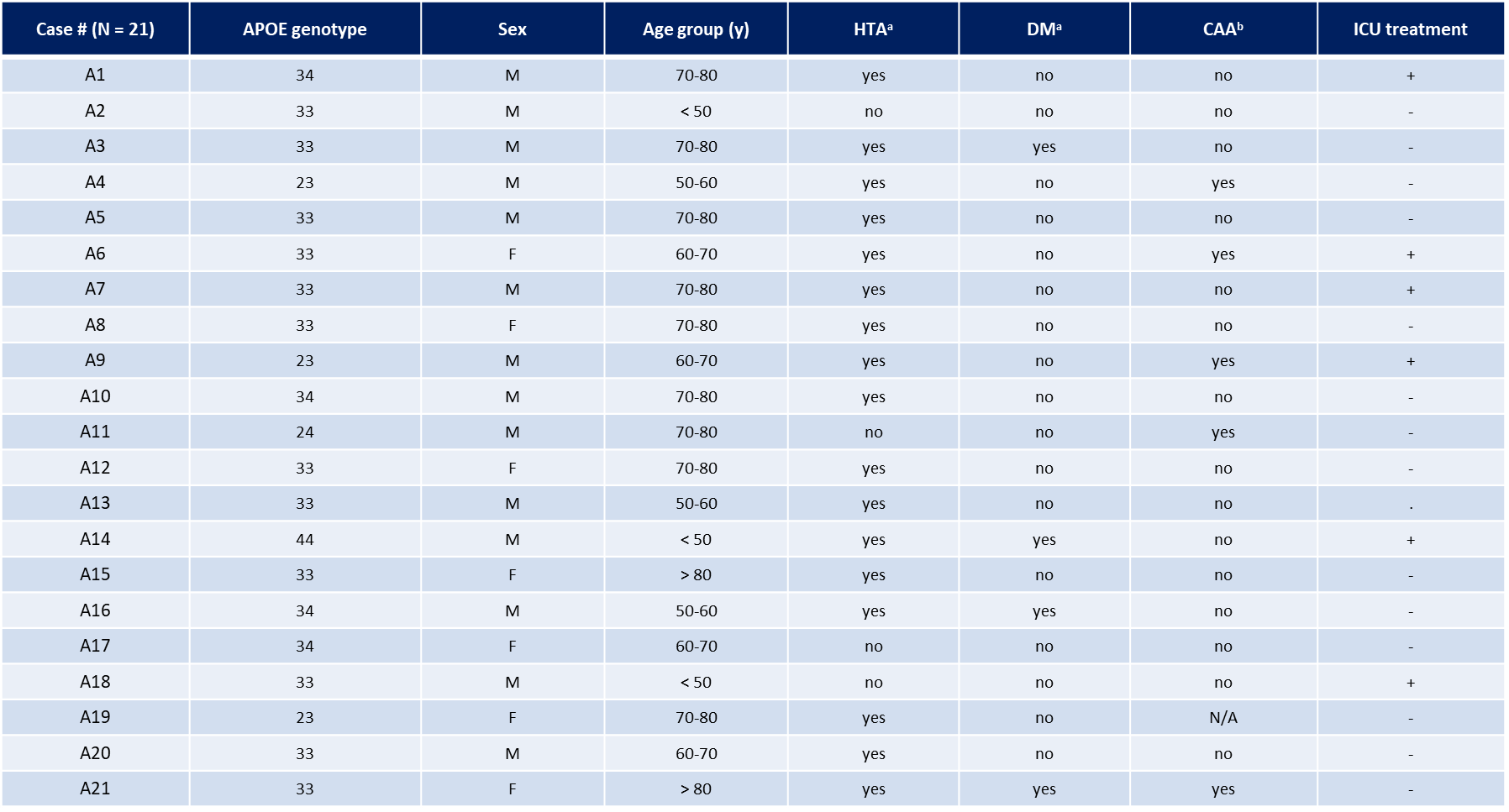


^a^ Diagnosis from electronic health record system. ^b^ Beta-amyloid staining of samples from cerebellum and frontal cortex; case declared positive if at least a single blood vessel stained positive.


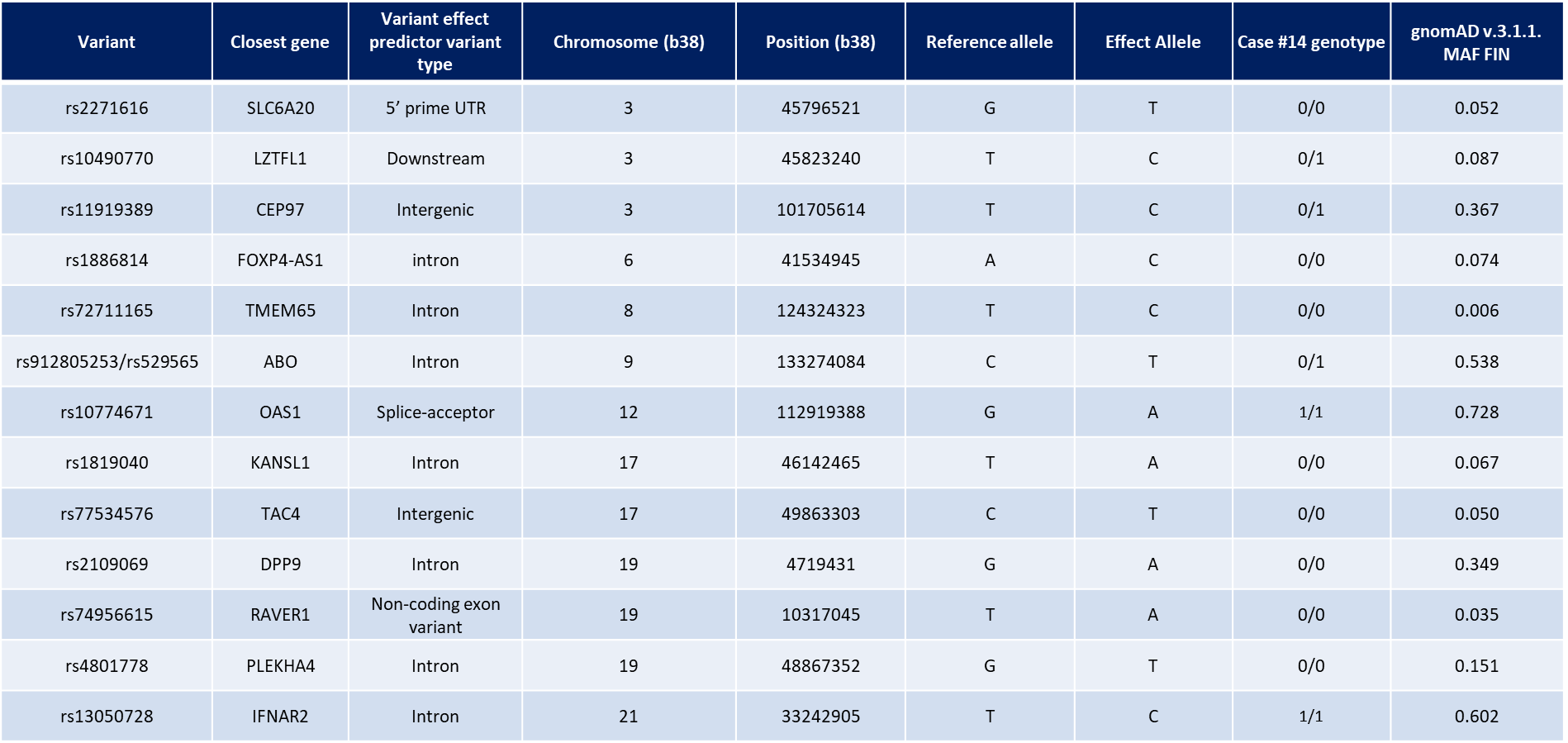
Supplementary Table 3. Top variant hits of COVID-19 WGS panel for AUTOPSY case #14 (A14)

^
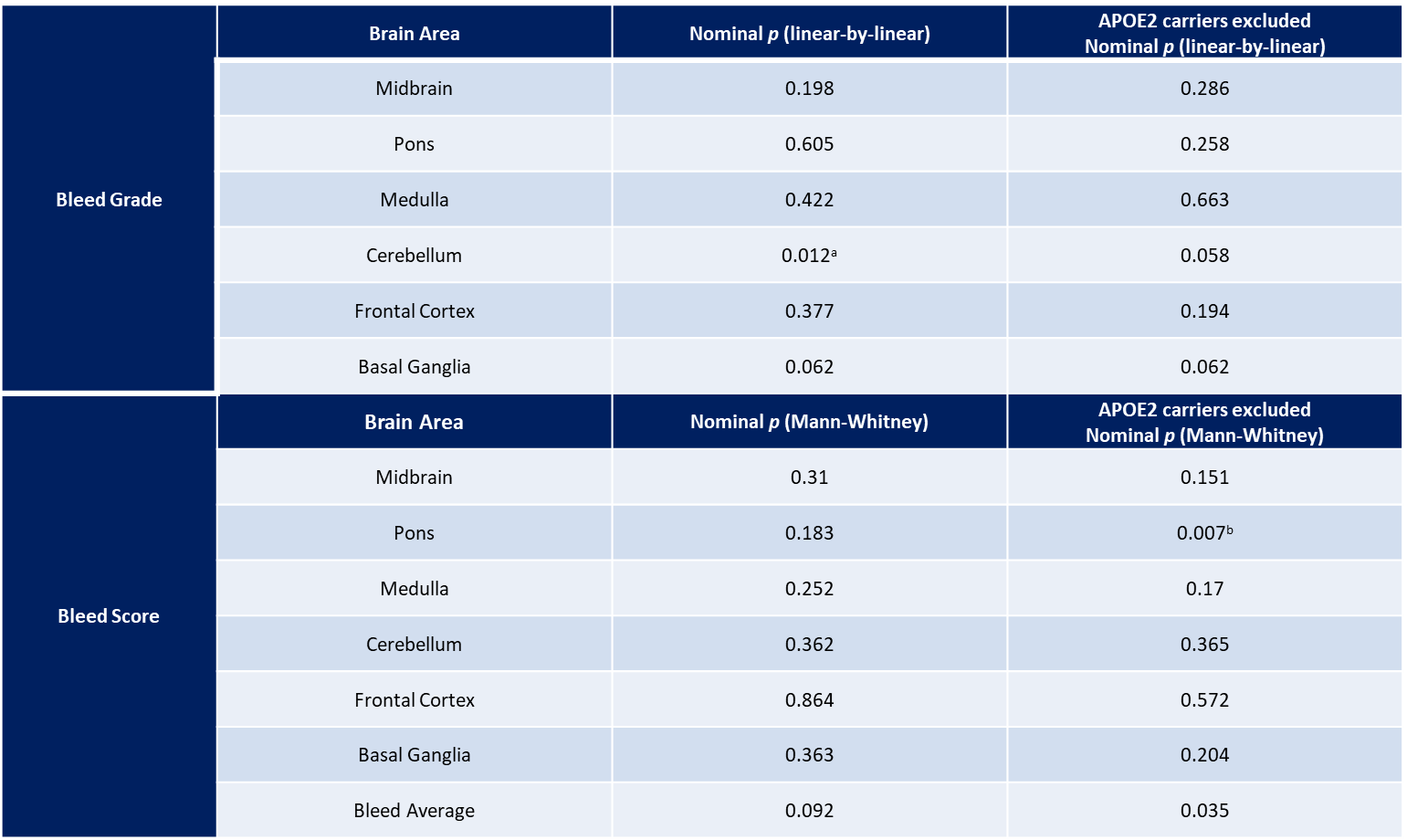
^Supplementary Table 4. Statistical analysis of perivascular haemorrhages

^a^Pearson’s chi-square test: Nominal *p* = 0.024; ^b^ Remaining statistically significant after Bonferroni’s correction for multiple testing

Supplementary Table 5a. Candidate gene studies on APOE and COVID-19
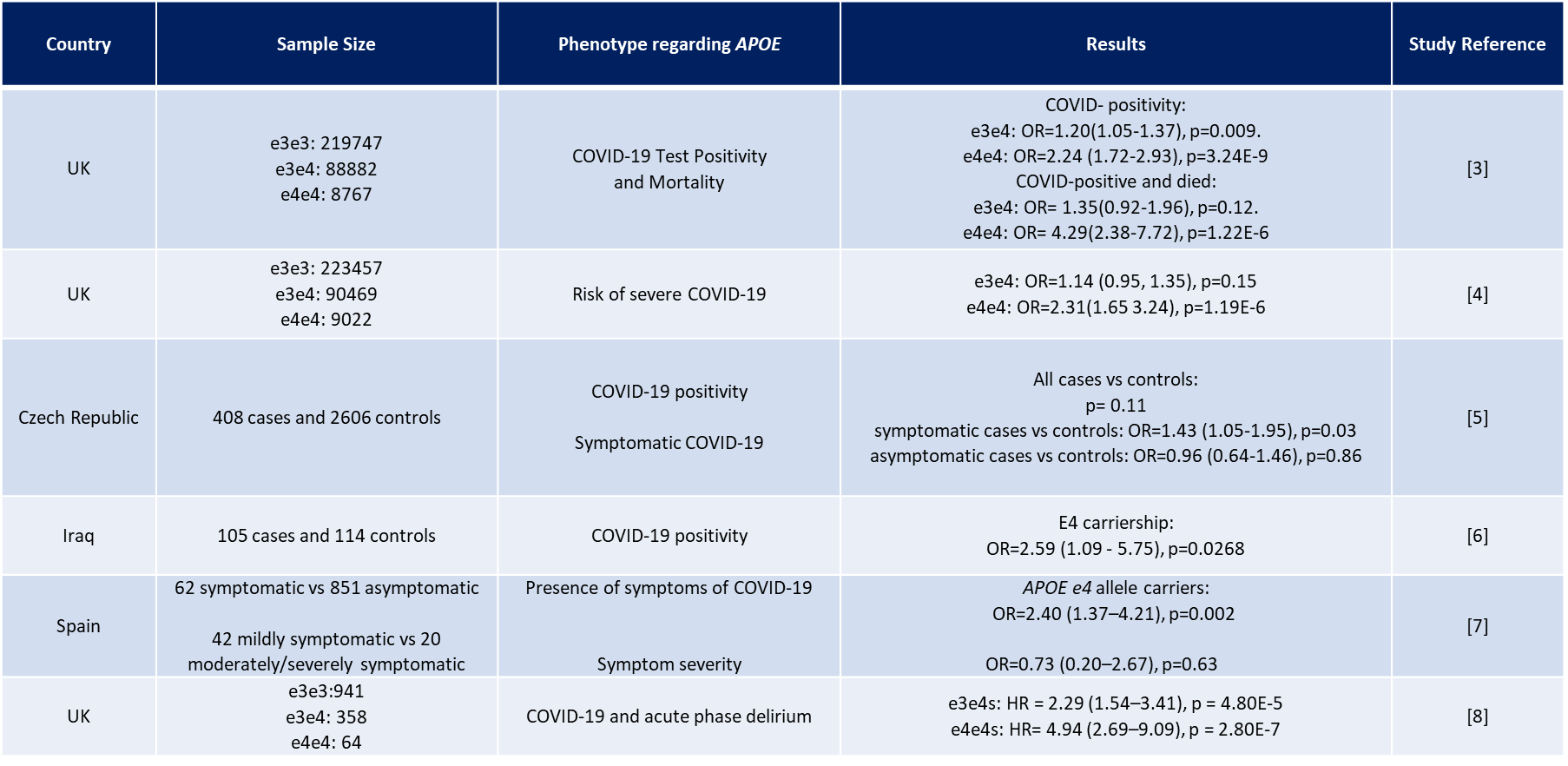


OR = Odds ratio (95% confidence interval); HR = Hazard ratio


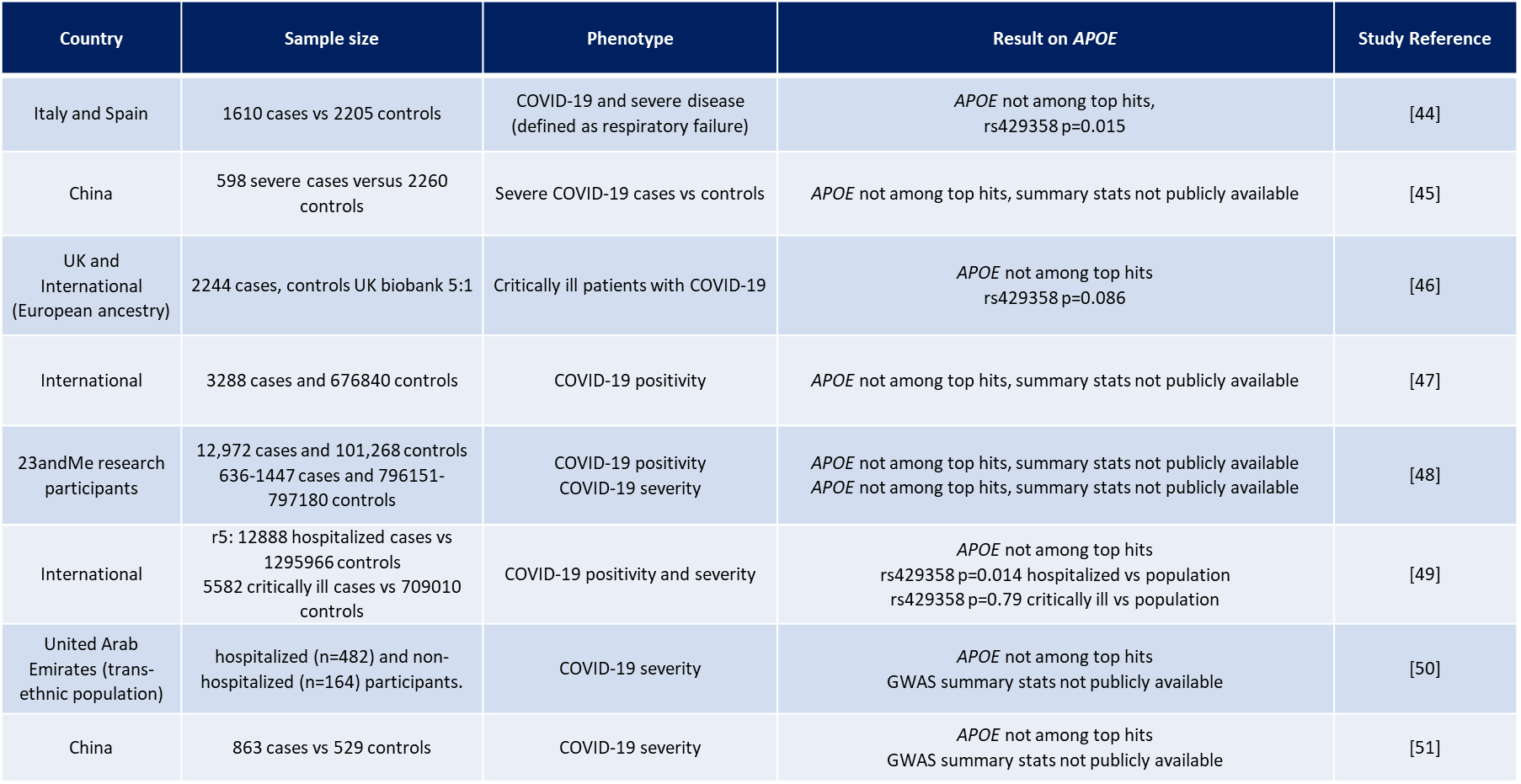
Supplementary Table 5b. Genome-wide association studies (GWAS) on COVID-19

OR = Odds ratio (95% confidence interval); HR = Hazard ratio

**Contributors of FinnGen**

**Steering Committee**

Aarno Palotie Institute for Molecular Medicine Finland, HiLIFE, University of Helsinki, Finland

Mark Daly Institute for Molecular Medicine Finland, HiLIFE, University of Helsinki, Finland

**Pharmaceutical companies**

Bridget Riley-Gills Abbvie, Chicago, IL, United States

Howard Jacob Abbvie, Chicago, IL, United States

Dirk Paul Astra Zeneca, Cambridge, United Kingdom

Heiko Runz Biogen, Cambridge, MA, United States

Sally John Biogen, Cambridge, MA, United States

George Okafo Boehringer Ingelheim, Ingelheim am Rhein, Germany

Nathan Lawless Boehringer Ingelheim, Ingelheim am Rhein, Germany

Robert Plenge Celgene, Summit, NJ, United States/Bristol Myers Squibb, New York, NY, United States

Joseph Maranville Celgene, Summit, NJ, United States/Bristol Myers Squibb, New York, NY, United States

Mark McCarthy Genentech, San Francisco, CA, United States

Julie Hunkapiller Genentech, San Francisco, CA, United States

Meg Ehm GlaxoSmithKline, Brentford, United Kingdom

Kirsi Auro GlaxoSmithKline, Brentford, United Kingdom

Simonne Longerich Merck, Kenilworth, NJ, United States

Caroline Fox Merck, Kenilworth, NJ, United States

Anders Mälarstig Pfizer, New York, NY, United States

Katherine Klinger Sanofi, Paris, France

Deepak Raipal Sanofi, Paris, France

Eric Green Maze Therapeutics, San Francisco, CA, United States

Robert Graham Maze Therapeutics, San Francisco, CA, United States

Robert Yang Janssen Biotech, Beerse, Belgium

Chris O´Donnell Novartis, Basel, Switzerland

**University of Helsinki & Biobanks**

Tomi Mäkelä HiLIFE, University of Helsinki, Finland, Finland

Jaakko Kaprio Institute for Molecular Medicine Finland, HiLIFE, Helsinki, Finland, Finland

Petri Virolainen Auria Biobank / University of Turku / Hospital District of Southwest Finland, Turku, Finland

Antti Hakanen Auria Biobank / University of Turku / Hospital District of Southwest Finland, Turku, Finland

Terhi Kilpi THL Biobank / The National Institute of Health and Welfare Helsinki, Finland

Markus Perola THL Biobank / The National Institute of Health and Welfare Helsinki, Finland

Jukka Partanen Finnish Red Cross Blood Service / Finnish Hematology Registry and Clinical Biobank, Helsinki, Finland

Anne Pitkäranta Helsinki Biobank / Helsinki University and Hospital District of Helsinki and Uusimaa, Helsinki

Juhani Junttila Northern Finland Biobank Borealis / University of Oulu / Northern Ostrobothnia Hospital District, Oulu, Finland

Raisa Serpi Northern Finland Biobank Borealis / University of Oulu / Northern Ostrobothnia Hospital District, Oulu, Finland

Tarja Laitinen Finnish Clinical Biobank Tampere **/** University of Tampere / Pirkanmaa Hospital District, Tampere, Finland

Veli-Matti Kosma Biobank of Eastern Finland / University of Eastern Finland / Northern Savo Hospital District, Kuopio, Finland

Jari Laukkanen Central Finland Biobank / University of Jyväskylä / Central Finland Health Care District, Jyväskylä, Finland

Marco Hautalahti FINBB - Finnish biobank cooperative

**Other Experts/ Non-Voting Members**

Outi Tuovila Business Finland, Helsinki, Finland

Raimo Pakkanen Business Finland, Helsinki, Finland

**Scientific Committee**

**Pharmaceutical companies**

Jeffrey Waring Abbvie, Chicago, IL, United States

Bridget Riley-Gillis Abbvie, Chicago, IL, United States

Ioanna Tachmazidou Astra Zeneca, Cambridge, United Kingdom

Chia-Yen Chen Biogen, Cambridge, MA, United States

Heiko Runz Biogen, Cambridge, MA, United States

Zhihao Ding Boehringer Ingelheim, Ingelheim am Rhein, Germany

Marc Jung Boehringer Ingelheim, Ingelheim am Rhein, Germany

Shameek Biswas Celgene, Summit, NJ, United States/Bristol Myers Squibb, New York, NY, United States

Rion Pendergrass Genentech, San Francisco, CA, United States

Julie Hunkapiller Genentech, San Francisco, CA, United States

Meg Ehm GlaxoSmithKline, Brentford, United Kingdom

David Pulford GlaxoSmithKline, Brentford, United Kingdom

Neha Raghavan Merck, Kenilworth, NJ, United States

Adriana Huertas-Vazquez Merck, Kenilworth, NJ, United States

Jae-Hoon Sul Merck, Kenilworth, NJ, United States

Anders Mälarstig Pfizer, New York, NY, United States

Xinli Hu Pfizer, New York, NY, United States

Katherine Klinger Sanofi, Paris, France

Matthias Gossel Sanofi, Paris, France

Robert Graham Maze Therapeutics, San Francisco, CA, United States

Eric Green Maze Therapeutics, San Francisco, CA, United States

Sahar Mozaffari Maze Therapeutics, San Francisco, CA, United States

Dawn Waterworth Janssen Research & Development, LLC, Spring House, PA, United States

Nicole Renaud Novartis, Basel, Switzerland

Ma´en Obeidat Novartis, Basel, Switzerland

**University of Helsinki & Biobanks**

Samuli Ripatti Institute for Molecular Medicine Finland, HiLIFE, Helsinki, Finland

Johanna Schleutker Auria Biobank / Univ. of Turku / Hospital District of Southwest Finland, Turku, Finland

Markus Perola THL Biobank / The National Institute of Health and Welfare Helsinki, Finland

Mikko Arvas Finnish Red Cross Blood Service / Finnish Hematology Registry and Clinical Biobank, Helsinki, Finland

Olli Carpén Helsinki Biobank / Helsinki University and Hospital District of Helsinki and Uusimaa, Helsinki

Reetta Hinttala Northern Finland Biobank Borealis / University of Oulu / Northern Ostrobothnia Hospital District, Oulu, Finland

Johannes Kettunen Northern Finland Biobank Borealis / University of Oulu / Northern Ostrobothnia Hospital District, Oulu, Finland

Arto Mannermaa Biobank of Eastern Finland / University of Eastern Finland / Northern Savo Hospital District, Kuopio, Finland

Katriina Aalto-Setälä Finnish Clinical Biobank Tampere **/** University of Tampere / Pirkanmaa Hospital District, Tampere, Finland

Mika Kähönen Finnish Clinical Biobank Tampere **/** University of Tampere / Pirkanmaa Hospital District, Tampere, Finland

Jari Laukkanen Central Finland Biobank / University of Jyväskylä / Central Finland Health Care District, Jyväskylä, Finland

Johanna Mäkelä FINBB - Finnish biobank cooperative

**Clinical Groups**

**Neurology Group**

Reetta Kälviäinen Northern Savo Hospital District, Kuopio, Finland

Valtteri Julkunen Northern Savo Hospital District, Kuopio, Finland

Hilkka Soininen Northern Savo Hospital District, Kuopio, Finland

Anne Remes Northern Ostrobothnia Hospital District, Oulu, Finland

Mikko Hiltunen Northern Savo Hospital District, Kuopio, Finland

Jukka Peltola Pirkanmaa Hospital District, Tampere, Finland

Minna Raivio Hospital District of Helsinki and Uusimaa, Helsinki, Finland

Pentti Tienari Hospital District of Helsinki and Uusimaa, Helsinki, Finland

Juha Rinne Hospital District of Southwest Finland, Turku, Finland

Roosa Kallionpää Hospital District of Southwest Finland, Turku, Finland

Juulia Partanen Institute for Molecular Medicine Finland, HiLIFE, University of Helsinki, Finland

Ali Abbasi Abbvie, Chicago, IL, United States

Adam Ziemann Abbvie, Chicago, IL, United States

Jeffrey Waring Abbvie, Chicago, IL, United States

Nizar Smaoui Abbvie, Chicago, IL, United States

Anne Lehtonen Abbvie, Chicago, IL, United States

Susan Eaton Biogen, Cambridge, MA, United States

Heiko Runz Biogen, Cambridge, MA, United States

Sanni Lahdenperä Biogen, Cambridge, MA, United States

Janet van Adelsberg Celgene, Summit, NJ, United States/ Bristol Myers Squibb, New York, NY, United States

Shameek Biswas Celgene, Summit, NJ, United States/ Bristol Myers Squibb, New York, NY, United States

Julie Hunkapiller Genentech, San Francisco, CA, United States

Natalie Bowers Genentech, San Francisco, CA, United States

Edmond Teng Genentech, San Francisco, CA, United States

Rion Pendergrass Genentech, San Francisco, CA, United States

Fanli Xu GlaxoSmithKline, Brentford, United Kingdom

David Pulford GlaxoSmithKline, Brentford, United Kingdom

Kirsi Auro GlaxoSmithKline, Brentford, United Kingdom

Laura Addis GlaxoSmithKline, Brentford, United Kingdom

John Eicher GlaxoSmithKline, Brentford, United Kingdom

Qingqin S Li Janssen Research & Development, LLC, Titusville, NJ 08560, United States

Karen He Janssen Research & Development, LLC, Spring House, PA, United States

Ekaterina Khramtsova Janssen Research & Development, LLC, Spring House, PA, United States

Neha Raghavan Merck, Kenilworth, NJ, United States

Kari Linden Pfizer, New York, NY, United States

**Gastroenterology Group**

Martti Färkkilä Hospital District of Helsinki and Uusimaa, Helsinki, Finland

Jukka Koskela Hospital District of Helsinki and Uusimaa, Helsinki, Finland

Sampsa Pikkarainen Hospital District of Helsinki and Uusimaa, Helsinki, Finland

Airi Jussila Pirkanmaa Hospital District, Tampere, Finland

Katri Kaukinen Pirkanmaa Hospital District, Tampere, Finland

Timo Blomster Northern Ostrobothnia Hospital District, Oulu, Finland

Mikko Kiviniemi Northern Savo Hospital District, Kuopio, Finland

Markku Voutilainen Hospital District of Southwest Finland, Turku, Finland

Mark Daly Institute for Molecular Medicine Finland, HiLIFE, University of Helsinki, Finland

Ali Abbasi Abbvie, Chicago, IL, United States

Graham Heap Abbvie, Chicago, IL, United States

Jeffrey Waring Abbvie, Chicago, IL, United States

Nizar Smaoui Abbvie, Chicago, IL, United States

Fedik Rahimov Abbvie, Chicago, IL, United States

Anne Lehtonen Abbvie, Chicago, IL, United States

Keith Usiskin Celgene, Summit, NJ, United States/ Bristol Myers Squibb, New York, NY, United States

Tim Lu Genentech, San Francisco, CA, United States

Natalie Bowers Genentech, San Francisco, CA, United States

Rion Pendergrass Genentech, San Francisco, CA, United States

Linda McCarthy GlaxoSmithKline, Brentford, United Kingdom

Amy Hart Janssen Research & Development, LLC, Spring House, PA, United States

Meijian Guan Janssen Research & Development, LLC, Spring House, PA, United States

Jason Miller Merck, Kenilworth, NJ, United States

Kirsi Kalpala Pfizer, New York, NY, United States

Melissa Miller Pfizer, New York, NY, United States

Xinli Hu Pfizer, New York, NY, United States

**Rheumatology Group**

Kari Eklund Hospital District of Helsinki and Uusimaa, Helsinki, Finland

Antti Palomäki Hospital District of Southwest Finland, Turku, Finland

Pia Isomäki Pirkanmaa Hospital District, Tampere, Finland

Laura Pirilä Hospital District of Southwest Finland, Turku, Finland

Oili Kaipiainen-Seppänen Northern Savo Hospital District, Kuopio, Finland

Johanna Huhtakangas Northern Ostrobothnia Hospital District, Oulu, Finland

Nina Mars Institute for Molecular Medicine Finland, HiLIFE, Helsinki, Finland

Ali Abbasi Abbvie, Chicago, IL, United States

Jeffrey Waring Abbvie, Chicago, IL, United States

Fedik Rahimov Abbvie, Chicago, IL, United States

Apinya Lertratanakul Abbvie, Chicago, IL, United States

Nizar Smaoui Abbvie, Chicago, IL, United States

Anne Lehtonen Abbvie, Chicago, IL, United States

David Close Astra Zeneca, Cambridge, United Kingdom

Marla Hochfeld Celgene, Summit, NJ, United States/ Bristol Myers Squibb, New York, NY, United States

Natalie Bowers Genentech, San Francisco, CA, United States

Rion Pendergrass Genentech, San Francisco, CA, United States

Jorge Esparza Gordillo GlaxoSmithKline, Brentford, United Kingdom

Kirsi Auro GlaxoSmithKline, Brentford, United Kingdom

Dawn Waterworth Janssen Research & Development, LLC, Spring House, PA, United States

Fabiana Farias Merck, Kenilworth, NJ, United States

Kirsi Kalpala Pfizer, New York, NY, United States

Nan Bing Pfizer, New York, NY, United States

Xinli Hu Pfizer, New York, NY, United States

**Pulmonology Group**

Tarja Laitinen Pirkanmaa Hospital District, Tampere, Finland

Margit Pelkonen Northern Savo Hospital District, Kuopio, Finland

Paula Kauppi Hospital District of Helsinki and Uusimaa, Helsinki, Finland

Hannu Kankaanranta University of Gothenburg, Gothenburg, Sweden/ Seinäjoki Central Hospital, Seinäjoki, Finland/ Tampere University, Tampere, Finland

Terttu Harju Northern Ostrobothnia Hospital District, Oulu, Finland

Riitta Lahesmaa Hospital District of Southwest Finland, Turku, Finland

Nizar Smaoui Abbvie, Chicago, IL, United States

Alex Mackay Astra Zeneca, Cambridge, United Kingdom

Glenda Lassi Astra Zeneca, Cambridge, United Kingdom

Susan Eaton Biogen, Cambridge, MA, United States

Steven Greenberg Celgene, Summit, NJ, United States/ Bristol Myers Squibb, New York, NY, United States

Hubert Chen Genentech, San Francisco, CA, United States

Rion Pendergrass Genentech, San Francisco, CA, United States

Natalie Bowers Genentech, San Francisco, CA, United States

Joanna Betts GlaxoSmithKline, Brentford, United Kingdom

Kirsi Auro GlaxoSmithKline, Brentford, United Kingdom

Rajashree Mishra GlaxoSmithKline, Brentford, United Kingdom

Majd Mouded Novartis, Basel, Switzerland

Debby Ngo Novartis, Basel, Switzerland

**Cardiometabolic Diseases Group**

Teemu Niiranen The National Institute of Health and Welfare Helsinki, Finland

Felix Vaura The National Institute of Health and Welfare Helsinki, Finland

Veikko Salomaa The National Institute of Health and Welfare Helsinki, Finland

Kaj Metsärinne Hospital District of Southwest Finland, Turku, Finland

Jenni Aittokallio Hospital District of Southwest Finland, Turku, Finland

Mika Kähönen Pirkanmaa Hospital District, Tampere, Finland

Jussi Hernesniemi Pirkanmaa Hospital District, Tampere, Finland

Juhani Junttila Northern Ostrobothnia Hospital District, Oulu, Finland

Markku Laakso Northern Savo Hospital District, Kuopio, Finland

Jussi Pihlajamäki Northern Savo Hospital District, Kuopio, Finland

Daniel Gordin Hospital District of Helsinki and Uusimaa, Helsinki, Finland

Juha Sinisalo Hospital District of Helsinki and Uusimaa, Helsinki, Finland

Marja-Riitta Taskinen Hospital District of Helsinki and Uusimaa, Helsinki, Finland

Tiinamaija Tuomi Hospital District of Helsinki and Uusimaa, Helsinki, Finland

Timo Hiltunen Hospital District of Helsinki and Uusimaa, Helsinki, Finland

Jari Laukkanen Central Finland Health Care District, Jyväskylä, Finland

Amanda Elliott Institute for Molecular Medicine Finland, HiLIFE, University of Helsinki, Finland / Broad Institute, Cambridge, MA, United States

Mary Pat Reeve Institute for Molecular Medicine Finland, HiLIFE, University of Helsinki, Finland

Sanni Ruotsalainen Institute for Molecular Medicine Finland, HiLIFE, University of Helsinki, Finland

Benjamin Challis Astra Zeneca, Cambridge, United Kingdom

Dirk Paul Astra Zeneca, Cambridge, United Kingdom

Keith Usiskin Celgene, Summit, NJ, United States/ Bristol Myers Squibb, New York, NY, United States

Julie Hunkapiller Genentech, San Francisco, CA, United States

Natalie Bowers Genentech, San Francisco, CA, United States

Rion Pendergrass Genentech, San Francisco, CA, United States

Audrey Chu GlaxoSmithKline, Brentford, United Kingdom

Kirsi Auro GlaxoSmithKline, Brentford, United Kingdom

Dermot Reilly Janssen Research & Development, LLC, Boston, MA, United States

Mike Mendelson Novartis, Boston, MA, United States

Jaakko Parkkinen Pfizer, New York, NY, United States

Melissa Miller Pfizer, New York, NY, United States

**Oncology Group**

Tuomo Meretoja Hospital District of Helsinki and Uusimaa, Helsinki, Finland

Heikki Joensuu Hospital District of Helsinki and Uusimaa, Helsinki, Finland

Olli Carpén Hospital District of Helsinki and Uusimaa, Helsinki, Finland

Lauri Aaltonen Hospital District of Helsinki and Uusimaa, Helsinki, Finland

Johanna Mattson Hospital District of Helsinki and Uusimaa, Helsinki, Finland

Eveliina Salminen Hospital District of Helsinki and Uusimaa, Helsinki, Finland

Annika Auranen Pirkanmaa Hospital District , Tampere, Finland

Peeter Karihtala Northern Ostrobothnia Hospital District, Oulu, Finland

Päivi Auvinen Northern Savo Hospital District, Kuopio, Finland

Klaus Elenius Hospital District of Southwest Finland, Turku, Finland

Johanna Schleutker Hospital District of Southwest Finland, Turku, Finland

Esa Pitkänen Institute for Molecular Medicine Finland, HiLIFE, University of Helsinki, Finland

Nina Mars Institute for Molecular Medicine Finland, HiLIFE, University of Helsinki, Finland

Mark Daly Institute for Molecular Medicine Finland, HiLIFE, University of Helsinki, Finland

Relja Popovic Abbvie, Chicago, IL, United States

Jeffrey Waring Abbvie, Chicago, IL, United States

Bridget Riley-Gillis Abbvie, Chicago, IL, United States

Anne Lehtonen Abbvie, Chicago, IL, United States

Jennifer Schutzman Genentech, San Francisco, CA, United States

Julie Hunkapiller Genentech, San Francisco, CA, United States

Natalie Bowers Genentech, San Francisco, CA, United States

Rion Pendergrass Genentech, San Francisco, CA, United States

Diptee Kulkarni GlaxoSmithKline, Brentford, United Kingdom

Kirsi Auro GlaxoSmithKline, Brentford, United Kingdom

Alessandro Porello Janssen Research & Development, LLC, Spring House, PA, United States

Andrey Loboda Merck, Kenilworth, NJ, United States

Heli Lehtonen Pfizer, New York, NY, United States

Stefan McDonough Pfizer, New York, NY, United States

Marika Crohns Sanofi, Paris, France

Sauli Vuoti Sanofi, Paris, France

**Opthalmology Group**

Kai Kaarniranta Northern Savo Hospital District, Kuopio, Finland

Joni A Turunen Hospital District of Helsinki and Uusimaa, Helsinki, Finland

Terhi Ollila Hospital District of Helsinki and Uusimaa, Helsinki, Finland

Hannu Uusitalo Pirkanmaa Hospital District, Tampere, Finland

Juha Karjalainen Institute for Molecular Medicine Finland, HiLIFE, University of Helsinki, Finland

Esa Pitkänen Institute for Molecular Medicine Finland, HiLIFE, University of Helsinki, Finland

Mengzhen Liu Abbvie, Chicago, IL, United States

Heiko Runz Biogen, Cambridge, MA, United States

Stephanie Loomis Biogen, Cambridge, MA, United States

Erich Strauss Genentech, San Francisco, CA, United States

Natalie Bowers Genentech, San Francisco, CA, United States

Hao Chen Genentech, San Francisco, CA, United States

Rion Pendergrass Genentech, San Francisco, CA, United States

**Dermatology Group**

Kaisa Tasanen Northern Ostrobothnia Hospital District, Oulu, Finland

Laura Huilaja Northern Ostrobothnia Hospital District, Oulu, Finland

Katariina Hannula-Jouppi Hospital District of Helsinki and Uusimaa, Helsinki, Finland

Teea Salmi Pirkanmaa Hospital District, Tampere, Finland

Sirkku Peltonen Hospital District of Southwest Finland, Turku, Finland

Leena Koulu Hospital District of Southwest Finland, Turku, Finland

Nizar Smaoui Abbvie, Chicago, IL, United States

Fedik Rahimov Abbvie, Chicago, IL, United States

Anne Lehtonen Abbvie, Chicago, IL, United States

David Choy Genentech, San Francisco, CA, United States

Rion Pendergrass Genentech, San Francisco, CA, United States

Dawn Waterworth Janssen Research & Development, LLC, Spring House, PA, United States

Kirsi Kalpala Pfizer, New York, NY, United States

Ying Wu Pfizer, New York, NY, United States

**Odontology Group**

Pirkko Pussinen Hospital District of Helsinki and Uusimaa, Helsinki, Finland

Aino Salminen Hospital District of Helsinki and Uusimaa, Helsinki, Finland

Tuula Salo Hospital District of Helsinki and Uusimaa, Helsinki, Finland

David Rice Hospital District of Helsinki and Uusimaa, Helsinki, Finland

Pekka Nieminen Hospital District of Helsinki and Uusimaa, Helsinki, Finland

Ulla Palotie Hospital District of Helsinki and Uusimaa, Helsinki, Finland

Maria Siponen Northern Savo Hospital District, Kuopio, Finland

Liisa Suominen Northern Savo Hospital District, Kuopio, Finland

Päivi Mäntylä Northern Savo Hospital District, Kuopio, Finland

Ulvi Gursoy Hospital District of Southwest Finland, Turku, Finland

Vuokko Anttonen Northern Ostrobothnia Hospital District, Oulu, Finland

Kirsi Sipilä Northern Ostrobothnia Hospital District, Oulu, Finland

Rion Pendergrass Genentech, San Francisco, CA, United States

**Women’s Health and Reproduction Group**

Hannele Laivuori Institute for Molecular Medicine Finland, HiLIFE, University of Helsinki, Finland

Venla Kurra Pirkanmaa Hospital District, Tampere, Finland

Laura Kotaniemi-Talonen Pirkanmaa Hospital District, Tampere, Finland

Oskari Heikinheimo Hospital District of Helsinki and Uusimaa, Helsinki, Finland

Ilkka Kalliala Hospital District of Helsinki and Uusimaa, Helsinki, Finland

Lauri Aaltonen Hospital District of Helsinki and Uusimaa, Helsinki, Finland

Varpu Jokimaa Hospital District of Southwest Finland, Turku, Finland

Johannes Kettunen Northern Ostrobothnia Hospital District, Oulu, Finland

Marja Vääräsmäki Northern Ostrobothnia Hospital District, Oulu, Finland

Outi Uimari Northern Ostrobothnia Hospital District, Oulu, Finland

Laure Morin-Papunen Northern Ostrobothnia Hospital District, Oulu, Finland

Maarit Niinimäki Northern Ostrobothnia Hospital District, Oulu, Finland

Terhi Piltonen Northern Ostrobothnia Hospital District, Oulu, Finland

Katja Kivinen Institute for Molecular Medicine Finland, HiLIFE, University of Helsinki, Finland

Elisabeth Widen Institute for Molecular Medicine Finland, HiLIFE, University of Helsinki, Finland

Taru Tukiainen Institute for Molecular Medicine Finland, HiLIFE, University of Helsinki, Finland

Mary Pat Reeve Institute for Molecular Medicine Finland, HiLIFE, University of Helsinki, Finland

Mark Daly Institute for Molecular Medicine Finland, HiLIFE, University of Helsinki, Finland

Liu Aoxing Institute for Molecular Medicine Finland, HiLIFE, University of Helsinki, Finland

Andrea Ganna Institute for Molecular Medicine Finland, HiLIFE, University of Helsinki, Finland

Niko Välimäki University of Helsinki, Helsinki, Finland

Eija Laakkonen University of Jyväskylä, Jyväskylä, Finland

Jaakko Tyrmi University of Oulu, Oulu, Finland / University of Tampere, Tampere, Finland

Heidi Silven University of Oulu, Oulu, Finland

Eeva Slitz University of Oulu, Oulu, Finland

Riikka Arffman University of Oulu, Oulu, Finland

Susanna Savukoski University of Oulu, Oulu, Finland

Triin Laisk Estonian biobank, Tartu, Estonia

Natalia Pujol Estonian biobank, Tartu, Estonia

Bridget Riley-Gillis Abbvie, Chicago, IL, United States

Mengzhen Liu Abbvie, Chicago, IL, United States

Rion Pendergrass Genentech, San Francisco, CA, United States

Janet Kumar GlaxoSmithKline, Brentford, United Kingdom

Kirsi Auro GlaxoSmithKline, Brentford, United Kingdom

**FinnGen Analysis working group**

Bridget Riley-Gillis Abbvie, Chicago, IL, United States

Reza Hammond Abbvie, Chicago, IL, United States

Fedik Rahimov Abbvie, Chicago, IL, United States

Sabah Kadri Abbvie, Chicago, IL, United States

Mengzhen Liu Abbvie, Chicago, IL, United States

Slavé Petrovski Astra Zeneca, Cambridge, United Kingdom

Eleonor Wigmore Astra Zeneca, Cambridge, United Kingdom

Adele Mitchell Biogen, Cambridge, MA, United States

Benjamin Sun Biogen, Cambridge, MA, United States

Ellen Tsai Biogen, Cambridge, MA, United States

Denis Baird Biogen, Cambridge, MA, United States

Paola Bronson Biogen, Cambridge, MA, United States

Ruoyu Tian Biogen, Cambridge, MA, United States

Stephanie Loomis Biogen, Cambridge, MA, United States

Yunfeng Huang Biogen, Cambridge, MA, United States

Till Andlauer Boehringer Ingelheim, Ingelheim am Rhein, Germany

Jatin Arora Boehringer Ingelheim, Ingelheim am Rhein, Germany

Ghadi Rai Boehringer Ingelheim, Ingelheim am Rhein, Germany

Zhihao Ding Boehringer Ingelheim, Ingelheim am Rhein, Germany

Lorenz Maier Boehringer Ingelheim, Ingelheim am Rhein, Germany

Karsten Quast Boehringer Ingelheim, Ingelheim am Rhein, Germany

Francisco Herruzo Boehringer Ingelheim, Ingelheim am Rhein, Germany

Daniel Lopez Boehringer Ingelheim, Ingelheim am Rhein, Germany

Marc Jung Boehringer Ingelheim, Ingelheim am Rhein, Germany

Boris Bartholdy Boehringer Ingelheim, Ingelheim am Rhein, Germany

Joseph Maranville Celgene, Summit, NJ, United States/ Bristol Myers Squibb, New York, NY, United States

Shameek Biswas Celgene, Summit, NJ, United States/ Bristol Myers Squibb, New York, NY, United States

Elmutaz Mohammed Celgene, Summit, NJ, United States/ Bristol Myers Squibb, New York, NY, United States

Samir Wadhawan Celgene, Summit, NJ, United States/ Bristol Myers Squibb, New York, NY, United States

Erika Kvikstad Celgene, Summit, NJ, United States/ Bristol Myers Squibb, New York, NY, United States

Diana Chang Genentech, San Francisco, CA, United States

Julie Hunkapiller Genentech, San Francisco, CA, United States

Tushar Bhangale Genentech, San Francisco, CA, United States

Natalie Bowers Genentech, San Francisco, CA, United States

Rion Pendergrass Genentech, San Francisco, CA, United States

Karen S King GlaxoSmithKline, Brentford, United Kingdom

Padhraig Gormley GlaxoSmithKline, Brentford, United Kingdom

Jimmy Liu GlaxoSmithKline, Brentford, United Kingdom

Karsten Sieber Janssen Research & Development, LLC, Spring House, PA, United States

Amy Hart Janssen Research & Development, LLC, Spring House, PA, United States

Meijian Guan Janssen Research & Development, LLC, Spring House, PA, United States

Shicheng Guo Janssen Research & Development, LLC, Spring House, PA, United States

Matt Brauer Maze Therapeutics, San Francisco, CA, United States

Jason Miller Merck, Kenilworth, NJ, United States

Fabiana Farias Merck, Kenilworth, NJ, United States

Jorge Del-Aguila Merck, Kenilworth, NJ, United States

Kirill Shkura Merck, Kenilworth, NJ, United States

Victor Neduva Merck, Kenilworth, NJ, United States

Huilei Xu Novartis, Basel, Switzerland

Amy Cole Novartis, Basel, Switzerland

Jonathan Chung Novartis, Basel, Switzerland

Jaison Jacob Novartis, Basel, Switzerland

Katrina de Lange Novartis, Basel, Switzerland

Jonas Zierer Novartis, Basel, Switzerland

Xing Chen Pfizer, New York, NY, United States

Åsa Hedman Pfizer, New York, NY, United States

Franck Auge Sanofi, Paris, France

Clement Chatelain Sanofi, Paris, France

Deepak Rajpal Sanofi, Paris, France

Dongyu Liu Sanofi, Paris, France

Katherine Call Sanofi, Paris, France

Tai-He Xia Sanofi, Paris, France

Mitja Kurki Institute for Molecular Medicine Finland, HiLIFE, University of Helsinki, Finland / Broad Institute, Cambridge, MA, United States

Samuli Ripatti Institute for Molecular Medicine Finland, HiLIFE, University of Helsinki, Finland

Mark Daly Institute for Molecular Medicine Finland, HiLIFE, University of Helsinki, Finland

Juha Karjalainen Institute for Molecular Medicine Finland, HiLIFE, University of Helsinki, Finland

Aki Havulinna Institute for Molecular Medicine Finland, HiLIFE, University of Helsinki, Finland

Juha Mehtonen Institute for Molecular Medicine Finland, HiLIFE, University of Helsinki, Finland

Priit Palta Institute for Molecular Medicine Finland, HiLIFE, University of Helsinki, Finland

Shabbeer Hassan Institute for Molecular Medicine Finland, HiLIFE, University of Helsinki, Finland

Pietro Della Briotta Parolo Institute for Molecular Medicine Finland, HiLIFE, University of Helsinki, Finland

Wei Zhou Broad Institute, Cambridge, MA, United States

Mutaamba Maasha Broad Institute, Cambridge, MA, United States

Shabbeer Hassan Institute for Molecular Medicine Finland, HiLIFE, University of Helsinki, Finland

Susanna Lemmelä Institute for Molecular Medicine Finland, HiLIFE, University of Helsinki, Finland

Manuel Rivas University of Stanford, Stanford, CA, United States

Aarno Palotie Institute for Molecular Medicine Finland, HiLIFE, University of Helsinki, Finland

Arto Lehisto Institute for Molecular Medicine Finland, HiLIFE, University of Helsinki, Finland

Andrea Ganna Institute for Molecular Medicine Finland, HiLIFE, University of Helsinki, Finland

Vincent Llorens Institute for Molecular Medicine Finland, HiLIFE, University of Helsinki, Finland

Hannele Laivuori Institute for Molecular Medicine Finland, HiLIFE, University of Helsinki, Finland

Taru Tukiainen Institute for Molecular Medicine Finland, HiLIFE, University of Helsinki, Finland

Mary Pat Reeve Institute for Molecular Medicine Finland, HiLIFE, University of Helsinki, Finland

Henrike Heyne Institute for Molecular Medicine Finland, HiLIFE, University of Helsinki, Finland

Nina Mars Institute for Molecular Medicine Finland, HiLIFE, University of Helsinki, Finland

Kimmo Palin University of Helsinki, Helsinki, Finland

Javier Garcia-Tabuenca University of Tampere, Tampere, Finland

Harri Siirtola University of Tampere, Tampere, Finland

Tuomo Kiiskinen Institute for Molecular Medicine Finland, HiLIFE, University of Helsinki, Finland

Jiwoo Lee Institute for Molecular Medicine Finland, HiLIFE, University of Helsinki, Finland / Broad Institute, Cambridge, MA, United States

Kristin Tsuo Institute for Molecular Medicine Finland, HiLIFE, University of Helsinki, Finland / Broad Institute, Cambridge, MA, United States

Amanda Elliott Institute for Molecular Medicine Finland, HiLIFE, University of Helsinki, Finland / Broad Institute, Cambridge, MA, United States

Kati Kristiansson THL Biobank / The National Institute of Health and Welfare Helsinki, Finland

Mikko Arvas Finnish Red Cross Blood Service / Finnish Hematology Registry and Clinical Biobank, Helsinki, Finland

Kati Hyvärinen Finnish Red Cross Blood Service, Helsinki, Finland

Jarmo Ritari Finnish Red Cross Blood Service, Helsinki, Finland

Olli Carpén Helsinki Biobank / Helsinki University and Hospital District of Helsinki and Uusimaa, Helsinki

Johannes Kettunen Northern Finland Biobank Borealis / University of Oulu / Northern Ostrobothnia Hospital District, Oulu, Finland

Katri Pylkäs University of Oulu, Oulu, Finland

Eeva Sliz University of Oulu, Oulu, Finland

Minna Karjalainen University of Oulu, Oulu, Finland

Tuomo Mantere Northern Finland Biobank Borealis / University of Oulu / Northern Ostrobothnia Hospital District, Oulu, Finland

Eeva Kangasniemi Finnish Clinical Biobank Tampere **/** University of Tampere / Pirkanmaa Hospital District, Tampere, Finland

Sami Heikkinen University of Eastern Finland, Kuopio, Finland

Arto Mannermaa Biobank of Eastern Finland / University of Eastern Finland / Northern Savo Hospital District, Kuopio, Finland

Eija Laakkonen University of Jyväskylä, Jyväskylä, Finland

Dhanaprakash Jambulingam University of Turku, Turku, Finland

Venkat Subramaniam Rathinakannan University of Turku, Turku, Finland

Nina Pitkänen Auria Biobank / University of Turku / Hospital District of Southwest Finland, Turku, Finland

**Biobank directors**

Lila Kallio Auria Biobank / University of Turku / Hospital District of Southwest Finland, Turku, Finland

Sirpa Soini THL Biobank / The National Institute of Health and Welfare Helsinki, Finland

Jukka Partanen Finnish Red Cross Blood Service / Finnish Hematology Registry and Clinical Biobank, Helsinki, Finland

Eero Punkka Helsinki Biobank / Helsinki University and Hospital District of Helsinki and Uusimaa, Helsinki

Raisa Serpi Northern Finland Biobank Borealis / University of Oulu / Northern Ostrobothnia Hospital District, Oulu, Finland

Sanna Siltanen Finnish Clinical Biobank Tampere **/** University of Tampere / Pirkanmaa Hospital District, Tampere, Finland

Veli-Matti Kosma Biobank of Eastern Finland / University of Eastern Finland / Northern Savo Hospital District, Kuopio, Finland

Teijo Kuopio Central Finland Biobank / University of Jyväskylä / Central Finland Health Care District, Jyväskylä, Finland

**FinnGen Teams**

**Administration**

Anu Jalanko Institute for Molecular Medicine Finland, HiLIFE, University of Helsinki, Finland

Huei-Yi Shen Institute for Molecular Medicine Finland, HiLIFE, University of Helsinki, Finland

Risto Kajanne Institute for Molecular Medicine Finland, HiLIFE, University of Helsinki, Finland

Mervi Aavikko Institute for Molecular Medicine Finland, HiLIFE, University of Helsinki, Finland

**Analysis**

Mitja Kurki Institute for Molecular Medicine Finland, HiLIFE, University of Helsinki, Finland / Broad Institute, Cambridge, MA, United States

Juha Karjalainen Institute for Molecular Medicine Finland, HiLIFE, University of Helsinki, Finland

Pietro Della Briotta Parolo Institute for Molecular Medicine Finland, HiLIFE, University of Helsinki, Finland

Arto Lehisto Institute for Molecular Medicine Finland, HiLIFE, University of Helsinki, Finland

Juha Mehtonen Institute for Molecular Medicine Finland, HiLIFE, University of Helsinki, Finland

Wei Zhou Broad Institute, Cambridge, MA, United States

Masahiro Kanai Broad Institute, Cambridge, MA, United States

Mutaamba Maasha Broad Institute, Cambridge, MA, United States

**Clinical Endpoint Development**

Hannele Laivuori Institute for Molecular Medicine Finland, HiLIFE, University of Helsinki, Finland

Aki Havulinna Institute for Molecular Medicine Finland, HiLIFE, University of Helsinki, Finland

Susanna Lemmelä Institute for Molecular Medicine Finland, HiLIFE, University of Helsinki, Finland

Tuomo Kiiskinen Institute for Molecular Medicine Finland, HiLIFE, University of Helsinki, Finland

L. Elisa Lahtela Institute for Molecular Medicine Finland, HiLIFE, University of Helsinki, Finland

**Communication**

Mari Kaunisto Institute for Molecular Medicine Finland, HiLIFE, University of Helsinki, Finland

**E-Science**

Elina Kilpeläinen Institute for Molecular Medicine Finland, HiLIFE, University of Helsinki, Finland

Timo P. Sipilä Institute for Molecular Medicine Finland, HiLIFE, University of Helsinki, Finland

Oluwaseun Alexander Dada Institute for Molecular Medicine Finland, HiLIFE, University of Helsinki, Finland

Awaisa Ghazal Institute for Molecular Medicine Finland, HiLIFE, University of Helsinki, Finland

Anastasia Shcherban Institute for Molecular Medicine Finland, HiLIFE, University of Helsinki, Finland

Rigbe Weldatsadik Institute for Molecular Medicine Finland, HiLIFE, University of Helsinki, Finland

**Genotyping**

Kati Donner Institute for Molecular Medicine Finland, HiLIFE, University of Helsinki, Finland

Timo P. Sipilä Institute for Molecular Medicine Finland, HiLIFE, University of Helsinki, Finland

**Sample Collection Coordination**

Anu Loukola Helsinki Biobank / Helsinki University and Hospital District of Helsinki and Uusimaa, Helsinki

**Sample Logistics**

Päivi Laiho THL Biobank / The National Institute of Health and Welfare Helsinki, Finland

Tuuli Sistonen THL Biobank / The National Institute of Health and Welfare Helsinki, Finland

Essi Kaiharju THL Biobank / The National Institute of Health and Welfare Helsinki, Finland

Markku Laukkanen THL Biobank / The National Institute of Health and Welfare Helsinki, Finland

Elina Järvensivu THL Biobank / The National Institute of Health and Welfare Helsinki, Finland

Sini Lähteenmäki THL Biobank / The National Institute of Health and Welfare Helsinki, Finland

Lotta Männikkö THL Biobank / The National Institute of Health and Welfare Helsinki, Finland

Regis Wong THL Biobank / The National Institute of Health and Welfare Helsinki, Finland

**Registry Data Operations**

Minna Brunfeldt THL Biobank / The National Institute of Health and Welfare Helsinki, Finland

Hannele Mattsson THL Biobank / The National Institute of Health and Welfare Helsinki, Finland

Kati Kristiansson THL Biobank / The National Institute of Health and Welfare Helsinki, Finland

Susanna Lemmelä Institute for Molecular Medicine Finland, HiLIFE, University of Helsinki, Finland

Sami Koskelainen THL Biobank / The National Institute of Health and Welfare Helsinki, Finland

Tero Hiekkalinna THL Biobank / The National Institute of Health and Welfare Helsinki, Finland

Teemu Paajanen THL Biobank / The National Institute of Health and Welfare Helsinki, Finland

**Sequencing Informatics**

Priit Palta Institute for Molecular Medicine Finland, HiLIFE, University of Helsinki, Finland

Kalle Pärn Institute for Molecular Medicine Finland, HiLIFE, University of Helsinki, Finland

Mart Kals Institute for Molecular Medicine Finland, HiLIFE, University of Helsinki, Finland

Shuang Luo Institute for Molecular Medicine Finland, HiLIFE, University of Helsinki, Finland

Vishal Sinha Institute for Molecular Medicine Finland, HiLIFE, University of Helsinki, Finland

**Trajectory**

Tarja Laitinen Pirkanmaa Hospital District, Tampere, Finland

Mary Pat Reeve Institute for Molecular Medicine Finland, HiLIFE, University of Helsinki, Finland

Marianna Niemi University of Tampere, Tampere, Finland

Harri Siirtola University of Tampere, Tampere, Finland

Javier Gracia-Tabuenca University of Tampere, Tampere, Finland

Mika Helminen University of Tampere, Tampere, Finland

Tiina Luukkaala University of Tampere, Tampere, Finland

Iida Vähätalo University of Tampere, Tampere, Finland

**Data protection officer**

Jyrki Pitkänen Institute for Molecular Medicine Finland, HiLIFE, University of Helsinki, Finland

**FINBB - Finnish biobank cooperative**

Marco Hautalahti

Johanna Mäkelä

Sarah Smith

Tom Southerington
